# Symmetry transitions during gating of the TRPV2 ion channel in lipid membranes

**DOI:** 10.7554/eLife.45779

**Published:** 2019-05-15

**Authors:** Lejla Zubcevic, Allen L Hsu, Mario J Borgnia, Seok-Yong Lee

**Affiliations:** 1 Department of BiochemistryDuke University School of MedicineDurhamUnited States; 2 Genome Integrity and Structural Biology LaboratoryNational Institute of Environmental Health Sciences, National Institutes of Health, Department of Health and Human ServicesResearch Triangle ParkUnited States; National Institute of Neurological Disorders and Stroke, National Institutes of HealthUnited States; The University of Texas at AustinUnited States

**Keywords:** TRP channel, *Oryctolagus cuniculus*, heat sensing ion channel, Ca2+ permeable channel, cryo-EM, ligand gated ion channel, Other

## Abstract

The Transient Receptor Potential Vanilloid 2 (TRPV2) channel is a member of the temperature-sensing thermoTRPV family. Recent advances in cryo-electronmicroscopy (cryo-EM) and X-ray crystallography have provided many important insights into the gating mechanisms of thermoTRPV channels. Interestingly, crystallographic studies of ligand-dependent TRPV2 gating have shown that the TRPV2 channel adopts two-fold symmetric arrangements during the gating cycle. However, it was unclear if crystal packing forces played a role in stabilizing the two-fold symmetric arrangement of the channel. Here, we employ cryo-EM to elucidate the structure of full-length rabbit TRPV2 in complex with the agonist resiniferatoxin (RTx) in nanodiscs and amphipol. We show that RTx induces two-fold symmetric conformations of TRPV2 in both environments. However, the two-fold symmetry is more pronounced in the native-like lipid environment of the nanodiscs. Our data offers insights into a gating pathway in TRPV2 involving symmetry transitions.

## Introduction

Transient Receptor Potential V (TRPV) channels are part of the larger TRP channel family which plays important roles in numerous physiological processes (Clapham et al., 2001). A subset of TRPV channels, including subtypes TRPV1-TRPV4, possess an intrinsic capability to sense heat and are therefore referred to as thermoTRPV channels (Cao et al., 2013a; Liu and Qin, 2016; Smith et al., 2002; Chung et al., 2003). TRPV1-TRPV4 are non-selective cation channels which play important physiological roles in sensing noxious heat (Bölcskei et al., 2010; Julius, 2013; Marwaha et al., 2016; Mitchell et al., 2014), maintaining cardiac structure (Katanosaka et al., 2014) and maintaining skin (Eytan et al., 2014; Imura et al., 2009; Kim et al., 2016), hair (Asakawa et al., 2006; Imura et al., 2007; Xiao et al., 2008) and bone physiology (Masuyama et al., 2008). A distinctive feature of TRPV1 and TRPV2 is their permeability to large organic cations (Chung et al., 2008), such as the cationic dye YO-PRO-1 and the sodium channel blocker QX-314. This feature has led to proposals to utilize these channels as conduits for delivering small molecules to intracellular targets (Puopolo et al., 2013). The non-conducting structures of TRPV1 and TRPV2 possess two restrictions, one at the selectivity filter (SF) and second one at the intracellular mouth of the pore (termed the common gate) (Liao et al., 2013; Zubcevic et al., 2016; Huynh et al., 2016). Both restrictions must open widely to accommodate the passage of large organic cations. However, the mechanism that enables such opening was long unclear. In order to study the permeation of both metal ions and large organic cations in TRPV2, we recently crystallized the rabbit resiniferatoxin (RTx)-sensitive (Zhang et al., 2016) TRPV2 channel with a truncation in the pore turret in the presence of the agonist RTx (Zubcevic et al., 2018a). This study led to the revelation that the binding of RTx leads to a two-fold symmetric (C2) opening at the selectivity filter that is wide enough to permeate YO-PRO-1. This unexpected result offered the first experimental evidence that the homotetrameric TRPV2 can adopt C2 symmetric conformations during the gating cycle. However, it was unclear if crystal contacts or the crystallization conditions (e.g. high concentration of Ca^2+^) played a role in stabilizing the C2 symmetry. In addition, the minimal TRPV2 construct used in the crystallographic study lacked the pore turret, a region that is not essential for function (Liao et al., 2013; Zubcevic et al., 2016; Zubcevic et al., 2018a; Yao et al., 2010) but had previously been shown to have a modulatory effect on gating in TRPV1 and TRPV2 (Jara-Oseguera et al., 2016; Dosey et al., 2019). It was uncertain if the absence of this region in our crystallographic study affected the symmetry of the channel.

**Figure 1.**
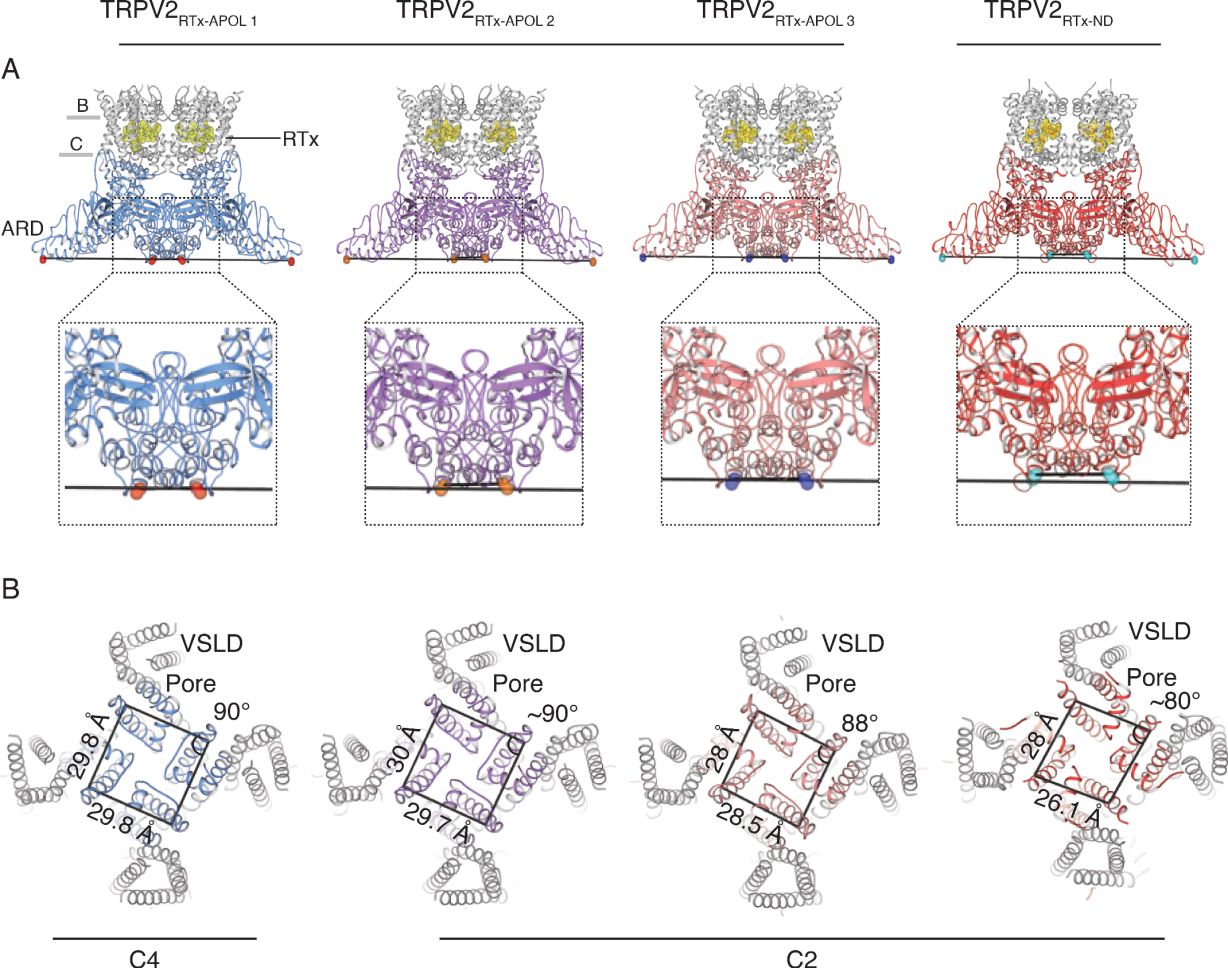
Overview of TRPV2_RTx-APOL_ and TRPV2_RTx-ND_ structures. (**A**) Orthogonal view of TRPV2_RTx-APOL 1-3_ and TRPV2_RTx-ND_ structures. TM domains are colored in grey and the cytoplasmic domains (ARD and C-terminal domain) are colored in blue, violet, salmon and red, respectively. RTx is shown in stick and sphere representation and colored in yellow. Lines drawn between diagonally opposite ARDs (residue E95, shown in red, orange, blue and cyan spheres, respectively) illustrate the relative position of ARDs in the tetramer. The close-up shows that the ankyrin repeats of diagonally opposing subunits in TRPV2_RTx-APOL 2_ and TRPV2_RTx-ND_ are positioned in different planes. (**B**) Top view of the channel (S5, S6 and PH are colored in blue, violet, salmon and red, respectively). Lines drawn between residues V620 in the S6 helix illustrate the symmetry within the pore domain. Distances and angles indicate the presence of two-fold symmetry.

In order to answer these questions and further study the role of two-fold symmetry in TRPV channel gating, we conducted cryo-electronmicroscopy (cryo-EM) studies of the full-length, RTx-sensitive rabbit TRPV2 (Zhang et al., 2016) channel reconstituted into nanodiscs and amphipol. We present four structures of the TRPV2/RTx complex, one obtained in nanodiscs (TRPV2_RTx-ND_) and three in amphipol (TRPV2_RTx-APOL 1-3_) determined to 3.8 Å, 2.9 Å, 3.3 Å and 4.2 Å resolution, respectively (Table 1, Figure 1). Our data shows that binding of RTx induces C2 symmetric conformations in TRPV2, but the extent of C2 symmetry depends on the environment in which the channel is reconstituted. C2 symmetry is particularly pronounced in the dataset collected from nanodisc-reconstituted TRPV2, which better approximates the physiological environment of the channel. Moreover, the data offers further insights into the allosteric coupling between the RTx-binding site and the activation gates in TRPV2, confirms the critical role of the S4-S5 linker π-helix (S4-S5_π-hinge_) in ligand-dependent gating of TRPV2, and provides a glimpse of the conformational landscape of TRPV2 gating.

**Figure 1—figure supplement 1.**
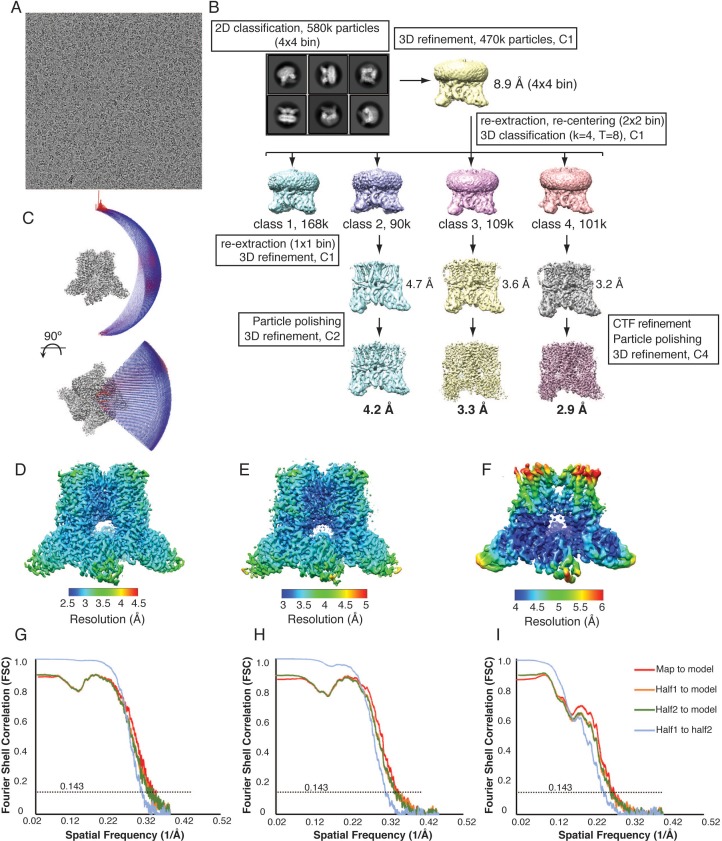
Cryo-EM data collection and processing, TRPV2_RTx-APOL_. (**A**) Representative micrograph from the TRPV2_RTx-APOL_ dataset. (**B**) 3D reconstruction workflow resulting in three distinct TRPV2_RTx-APOL_ structures. (**C**) Euler plot distribution. Red regions signify the best represented views. (**D–F**) Local resolution estimates calculated in RELION for TRPV2_RTx-APOL 1 _(**D**), TRPV2_RTx-APOL 2 _(**E**), TRPV2_RTx-APOL 3 _(**F**). (**G-I**) FSC curves calculated between the half maps (blue), atomic model and the final map (red), and between the model and each half-map (orange and green) for TRPV2_RTx-APOL 1_(**G**), TRPV2_RTx-APOL 2_(**H**), TRPV2_RTx-APOL 3_(**I**).

**Figure 1—figure supplement 2.**
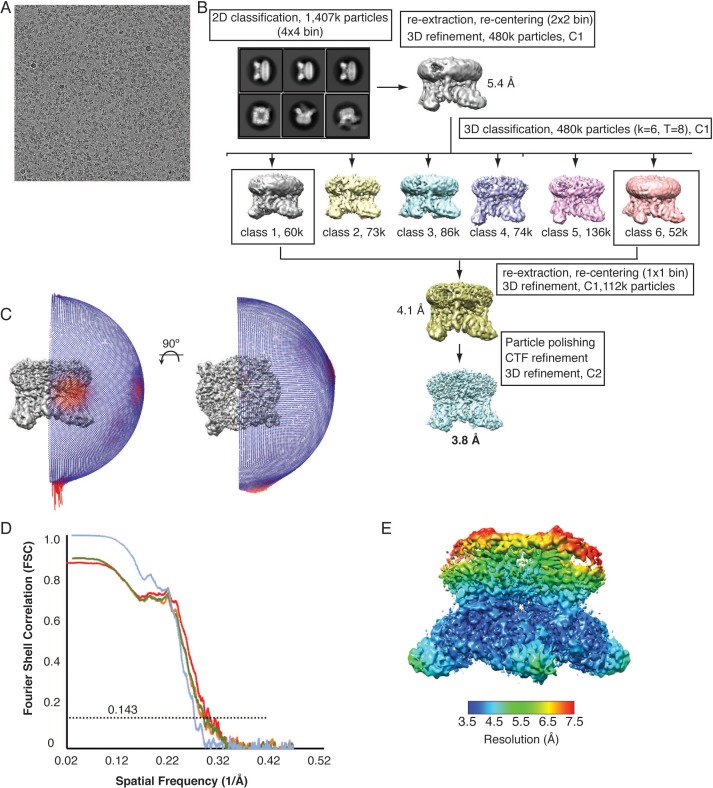
Cryo-EM data collection and processing, TRPV2_RTx-ND_. (**A**) Representative micrograph from the collected TRPV2_RTx-ND_ dataset. (**B**) 3D reconstruction workflow. (**C**) Euler distribution plot. Red regions indicate the best represented views. (**D**) FSC curves calculated between the half maps (blue), atomic model and the final map (red), and between the model and each half-map (orange and green). (**E**) Local resolution estimate, calculated in RELION.

**Figure 1—figure supplement 3.**
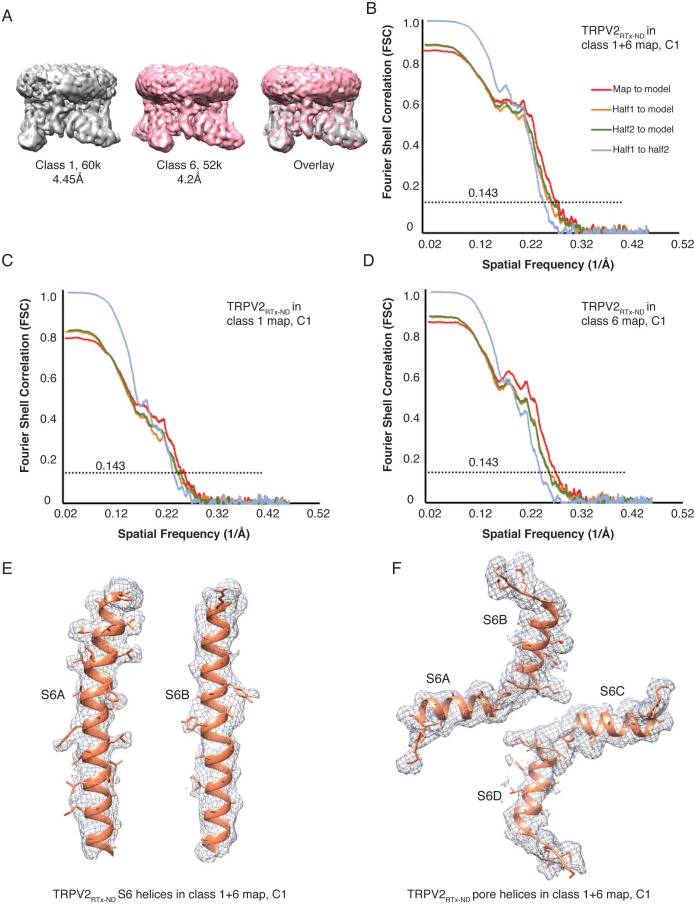
C2 symmetry in the TRPV2_RTx-ND_. (**A**) Classes 1 and 6 from the 3D classification of partcles from the TRPV2_RTx-ND_ dataset (Figure 1—figure supplement 2) refined individually with no symmetry imposed. (**B**) To evaluate the C2 symmetry in TRPV2_RTx-ND_, FSC curves were calculated between the non-symmetrized (C1) combined class 1 and 6 map. This map contains the same particles as the final map in Figure 1—figure supplement 2. Half maps (blue), atomic model and the full map (red), and between the model and each half-map (orange and green). (**C**) Fit of TRPV2_RTx-ND_ coordinates into the refined, non-symmetrized map of class 1. FSC curves between half maps (blue), atomic model and the full map (red), and between the model and each half-map (orange and green). (**D**) Fit of TRPV2_RTx-ND_ coordinates into the refined, non-symmetrized map of class 6. FSC curves between half maps (blue), atomic model and the full map (red), and between the model and each half-map (orange and green). (**E–F**) Electron density around the S6 helices (**E**) and the pore helices (**F**) in the combined class 1 and class 6 map with no symmetry imposed. The density is contoured at level 0.01 and radius 2.

**Figure 1—figure supplement 4.**
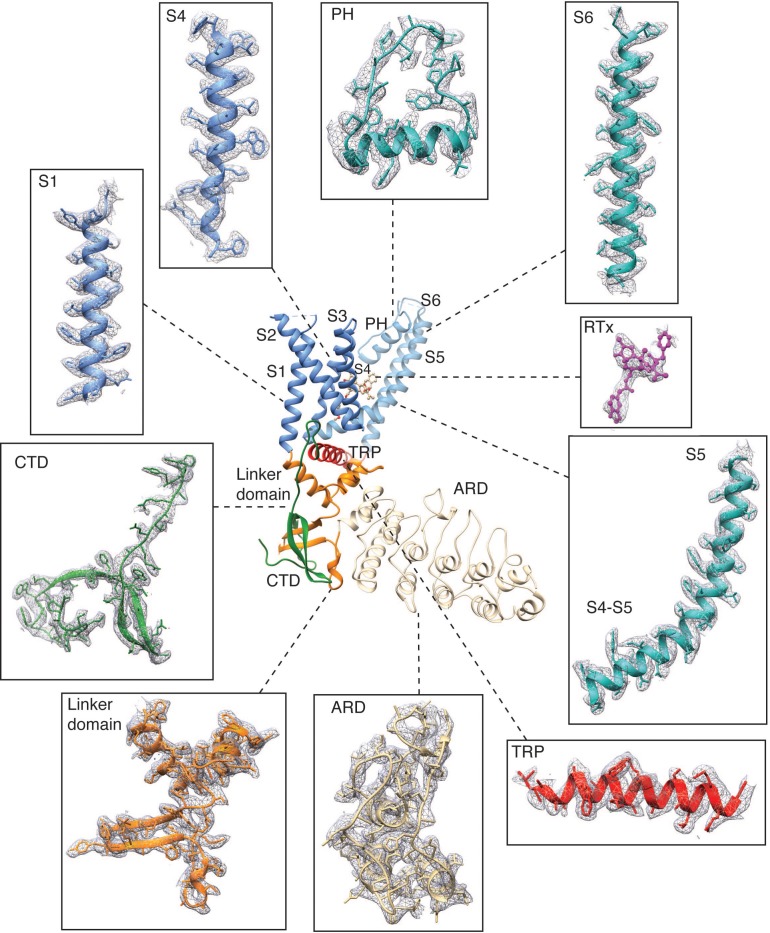
Representative electron densities in the TRPV2_RTx-APOL 1_ cryo-EM map. Densities are contoured at level 0.06 and radius 2.

**Figure 1—figure supplement 5.**
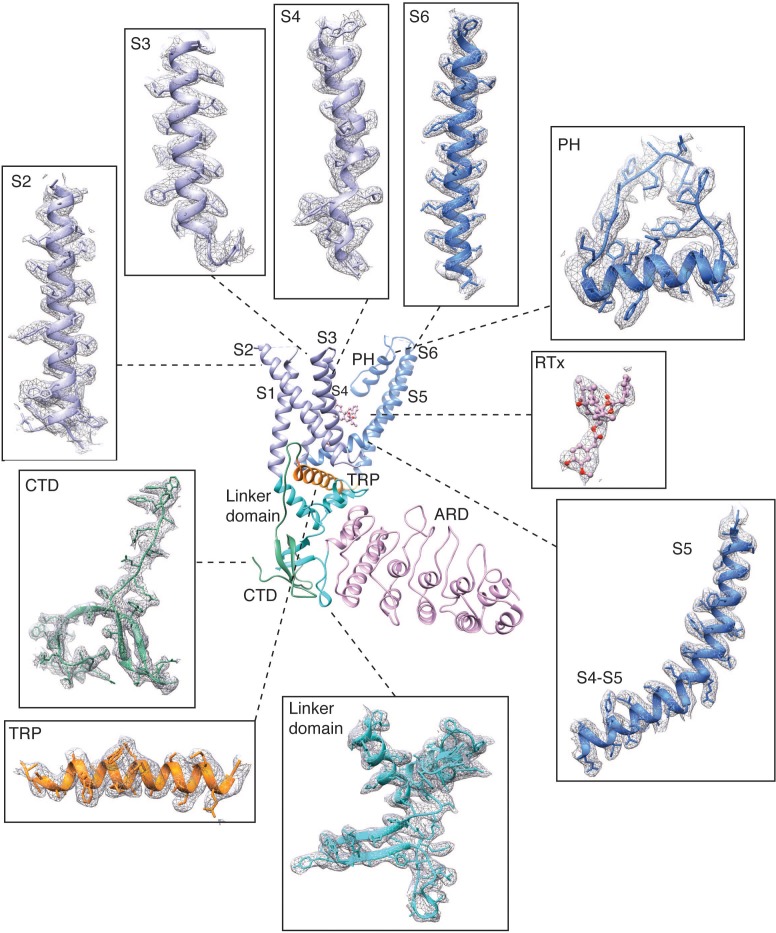
Representative electron densities in the TRPV2_RTx-APOL 2_ cryo-EM map. Densities are contoured at level 0.06 and radius 2.

**Figure 1—figure supplement 6.**
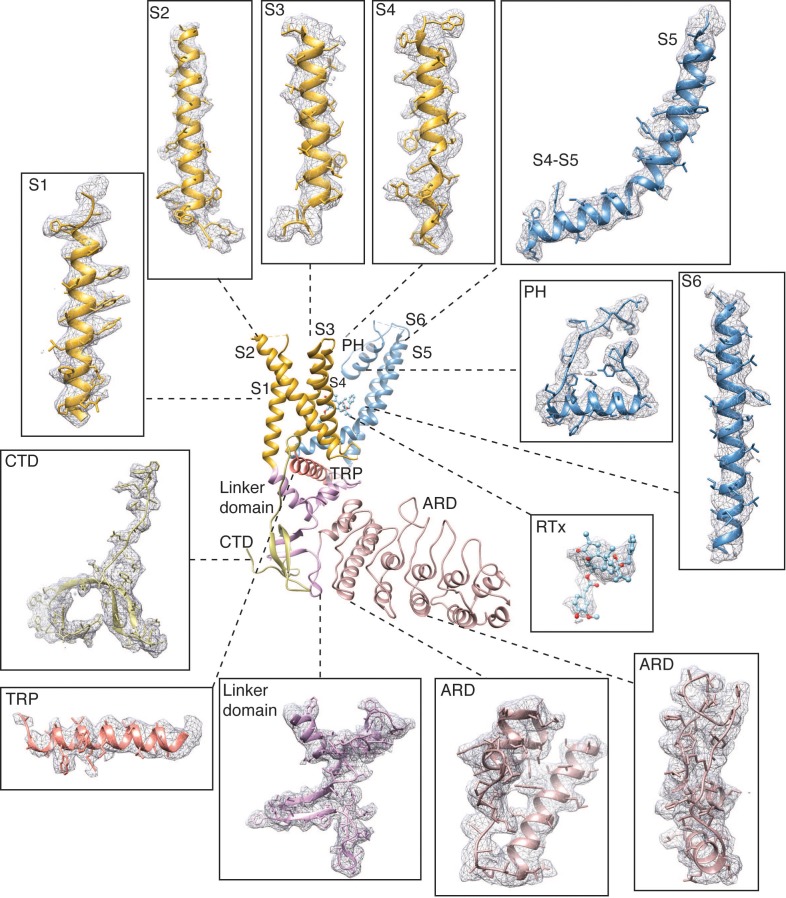
Representative electron densities in the TRPV2_RTx-APOL 3_ cryo-EM map. Densities are contoured at level 0.02 and radius 2.

**Figure 1—figure supplement 7.**
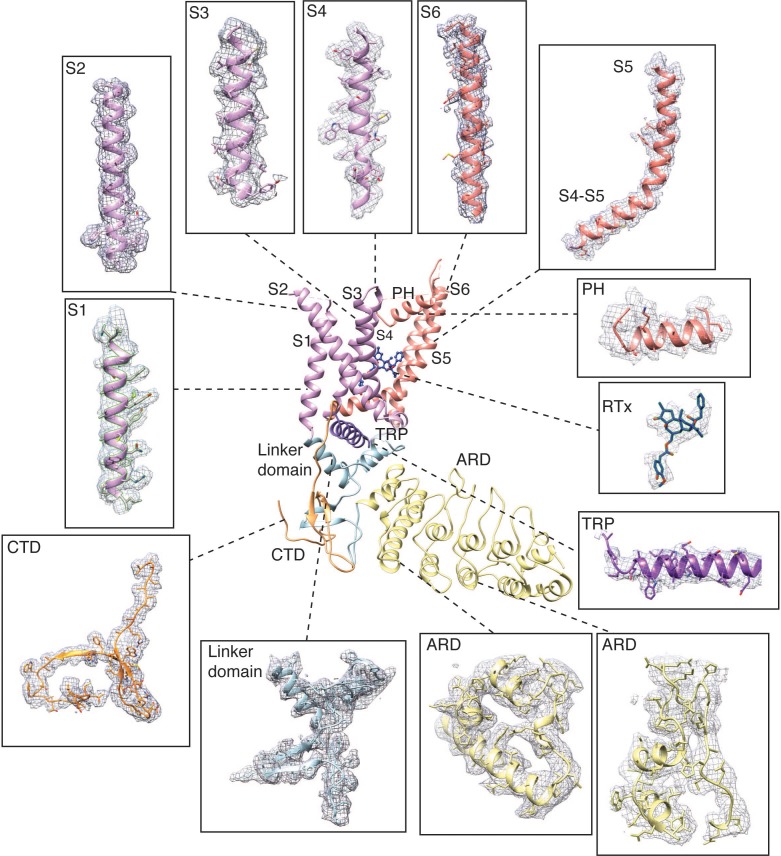
Representative electron densities in the TRPV2_RTx-ND_ cryo-EM map. Densities are contoured at level 0.01–0.03 and radius 2.

**Table 1.** Data collection and refinement statistics

Data collection and processing	TRPV2_RTx-ND_	TRPV2_RTx-APOL 1_	TRPV2_RTx-APOL 2_	TRPV2_RTx-APOL 3_
Electron microscope	Titan Krios	Titan Krios		
Electron detector	Falcon III	Falcon III		
Magnification	75,000x	75,000x		
Voltage (kV)	300	300		
Electron exposure (e–/Å^2^)	42	42		
Defocus range (μm)	−1.25 to −3.0	−1.25 to −3.0		
Pixel size (Å)	1.08	1.08		
Detector	Counting	Counting		
Total extracted particles (no.)	1,407,292	580,746		
Refined particles (no.)	482,602	470,760		
**Reconstruction**				
Final particles (no.)	112,622	101,570	109,623	90,862
Symmetry imposed	C2	C4	C2	C2
Nominal Resolution (Å)	3.8	2.9	3.3	4.19
FSC 0.143 (masked/unmasked)	3.7/3.9	2.9/3.05	3.2/3.5	4.0/4.3
Map sharpening *B* factor (Å^2^)	−30	−78	−92	−133
**Refinement**				
**Model composition** Non-hydrogen atoms Protein residues Ligands	16,878 2396 RTx: 4	18,236 2404 RTx: 4	18,452 2440 RTx: 4	17,548 2440 RTx: 4
**Validation** MolProbity score Clashscore Poor rotamers (%)	1.39 4 0	1.11 1.9 0	1.28 2.7 0	1.37 2.7 0
**Ramachandran plot**				
Favored (%) Allowed (%) Disallowed (%)	96.5 3.5 0	97.1 2.9 0	96.6 3.4 0	95.5 4.5 0

## Results

In order to capture the RTx-induced gating transitions in the rabbit TRPV2 channel, we conducted cryo-EM studies of the TRPV2/RTx complex reconstituted into amphipol (TRPV2_RTx-APOL_) and nanodiscs (TRPV2_RTx-ND_). Amphipols (Zoonens and Popot, 2014) have been a useful tool in structural studies of membrane proteins, and especially TRP channels (Liao et al., 2013; Zubcevic et al., 2016; Cao et al., 2013b; Paulsen et al., 2015; Yoo et al., 2018; Hirschi et al., 2017; Zubcevic et al., 2018b). Indeed, Amphipol A8-35 enabled the very first structural determination of the TRPV2 channel (Zubcevic et al., 2016). Nanodiscs, on the other hand, represent the closest in vitro approximation to the native lipid membranes used in structural studies (Denisov and Sligar, 2016). The data was processed using RELION (Scheres, 2012) (Materials and methods), with no symmetry imposed during particle classification and 3D reconstruction in order to avoid obscuring any classes with lower symmetry (C1 and C2) that might exist in the sample. Symmetry was only imposed in the last step of the refinement and only if the 3D reconstructions showed clear two-fold (C2) or four-fold (C4) symmetry (Figure 1—figure supplements 1–3). Classification of the TRPV2_RTx-APOL_ sample revealed the presence of four classes: one low-resolution (~7 Å) class, which was excluded from further analysis, and three higher resolution classes which are representative of three different conformations. These include one C4 symmetric and two distinct C2 symmetric classes refined to 2.9 Å, 3.3 Å and 4.2 Å, respectively (Figure 1, Figure 1—figure supplement 1). By contrast, 3D classification of the TRPV2_RTx-ND_ converged on a single C2 symmetric conformation resolved to 3.8 Å (Figure 1, Figure 1—figure supplements 2–3). All four maps were of sufficient quality to enable placement of individual structural motifs with confidence (Figure 1—figure supplements 4–7) and the models for all four structures were built to good overall geometry (Table 1).

### The transmembrane domains of TRPV2_RTx-APOL_ are trapped in a closed conformation

Unexpectedly, the transmembrane domains (TM) of the three structures obtained from amphipol-reconstituted TRPV2, TRPV2_RTx-APOL 1-3_, show similarity to our previously solved cryo-EM structure of TRPV2 in its apo form (Zubcevic et al., 2016) (TRPV2_APO_) and adopt non-conducting conformations (Figure 2—figure supplement 1). While fully bound to RTx, the TM domains of TRPV2_RTx-APOL 1_ and TRPV2_RTx-APOL 2_ structures largely retain C4 symmetry (Figure 1 and Figure 3—figure supplement 1). However, the TMs of TRPV2_RTx-APOL 3_ exhibit a slight departure from C4 symmetry in the pore (Figure 3—figure supplement 2). The effects of RTx on the TRPV2_RTx-APOL_ are particularly obvious in the ankyrin repeat domains (ARD) of the two-fold symmetric TRPV2_RTx-APOL 2_ and TRPV2_RTx-APOL 3_ which display pronounced broken symmetry and a range of rotational states (Figure 1A, Figure 3—figure supplements 1–3).

In order to determine the effect of RTx on the TRPV2_RTx-APOL_ sample, we aligned TRPV2_RTx-APOL 1_ with TRPV2_APO_. The transmembrane helices S1-S6 of the two channels aligned remarkably well (Cα R.M.S.D = 0.86) (Figure 3—figure supplement 1). However, RTx binding induces a 5° clockwise rotation of the ARD when viewed from the extracellular space and a ~ 10 Å lateral widening of the cytoplasmic assembly (Figure 3—figure supplement 1). In addition, RTx causes a conformational change in the S4-S5 linker (Figure 3—figure supplement 1), as well as a displacement of the TRP domain (Figure 3—figure supplement 1). The conformational change in the S4-S5 linker is caused by the introduction of a π-helical turn at the junction of the S4-S5 linker and the S5 helix in the TRPV2_RTx-APOL 1_ structure (S4-S5_π-hinge_), which is absent in TRPV2_APO_ (Figure 3—figure supplements 1 and 4). This observation concurs with our previous finding that RTx binding elicits a conformational change in the S4-S5 linker, and that the S4-S5_π-hinge_ is critical for ligand-dependent gating in TRPV2 (Zubcevic et al., 2018a). In TRPV2_RTx-APOL 3,_ slight C2 symmetry is observed in the TM domains and is evident in the SF, PH and the S4-S5 linker (Figure 3—figure supplement 2). Nevertheless, the RTx-induced conformational changes in the S4-S5 linker are not efficiently propagated to the TM in the TRPV2_RTx-APOL_ structures, and they fail to open either of the two restrictions in the pore (Figure 2—figure supplement 1). Instead, RTx only effects changes in its immediate binding site above the S4-S5 linker and in the parts of the channel not bound by amphipol, strongly suggesting that the polymer constricts the TM and prevents conformational changes at the S4-S5 linker and the ARD from propagating to the TM domain. The fact that the TRPV2/RTx complex is stabilized in multiple distinct closed states with different arrangements of the ARD assembly (Figure 1, Figure 3—figure supplements 1–3) suggests that the conformational changes in the ARD might represent low-energy, pre-open states that can be achieved without substantial changes in the TM domains.

Interestingly, metal ions are not visualized in the pores of any of the TRPV2_RTx-APOL_ structures, despite the high resolutions obtained in this study. Whether this is the result of cryo-EM experimental conditions is unclear, but thus far metal ions occupying the SF and the pores of thermoTRPV channels have only been captured in structures obtained by X-ray crystallography (Zubcevic et al., 2018a).

### RTx induces a break in symmetry in TRPV2_RTx-ND_

In stark contrast to the amphipol-reconstituted channel, RTx binding induces C2 symmetry in the nanodisc-reconstituted TRPV2 which extends throughout the channel. The symmetry of the TRPV2_RTx-ND_ map was assessed both visually, and by the *Map Symmetry* function in Phenix which yielded a CC = 0.9 and score of 1.28 for C2 symmetry. For comparison, C4 symmetry yielded a lower correlation coefficient (CC = 0.8). To further confirm the correctness of the symmetry assignment, we evaluated the fit of the TRPV2_RTx-ND_ model built into the C2 symmetric map to the non-symmetrized C1 map (Figure 1—figure supplement 3). In addition, we evaluated the fit of the TRPV2_RTx-ND_ model to the individually refined non-symmetrized classes 1 and 6, which constitute the TRPV2_RTx-ND_ map (Figure 1—figure supplement 3). All FSC curves indicate that the two-fold symmetric model fits well into the C1 maps and the density of the C1 symmetric TRPV2_RTx-ND_ map supports the model (Figure 1—figure supplement 3), showing that two-fold symmetry is truly present in the TRPV2_RTx-ND_ sample.

**Figure 2.**
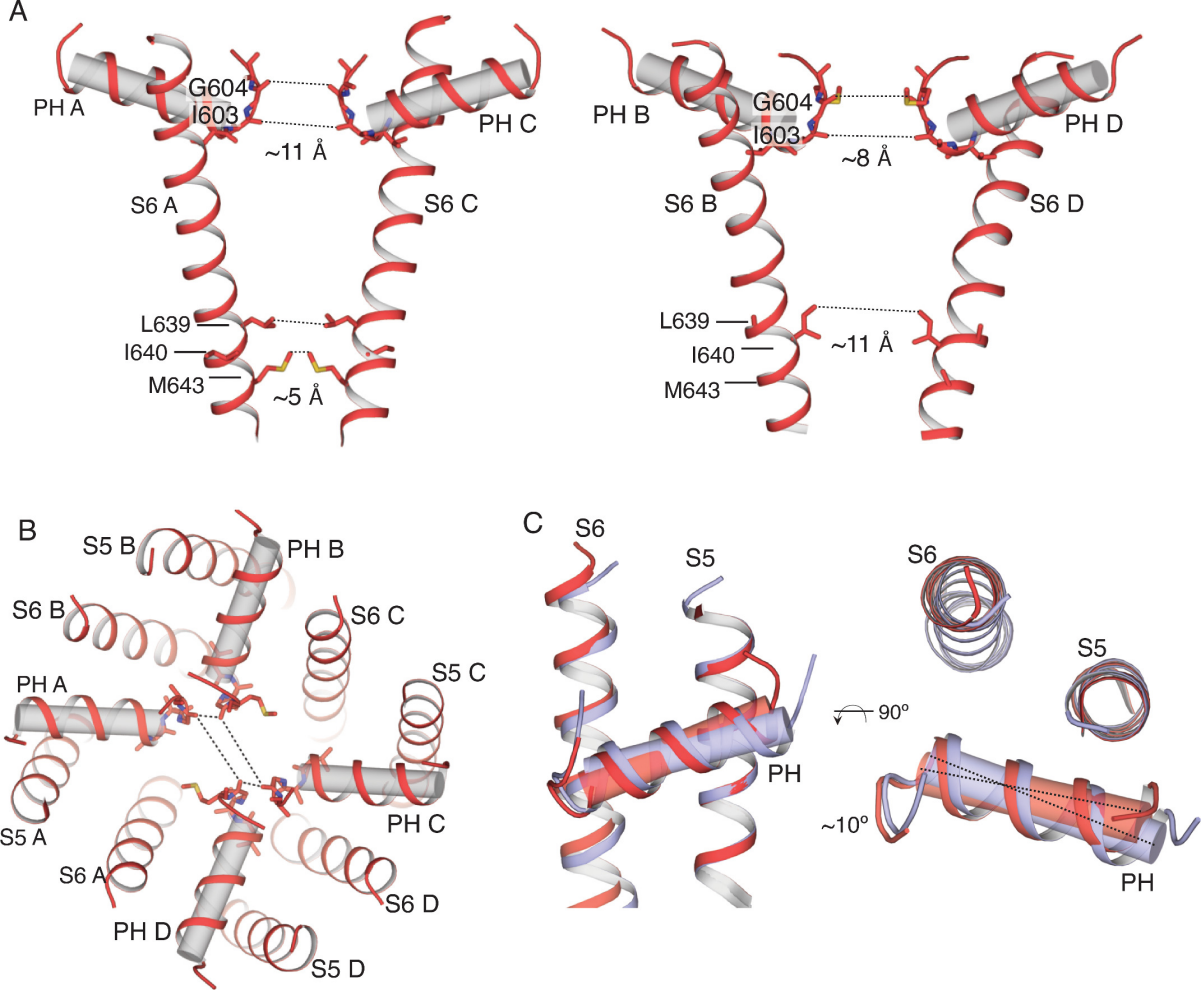
Overview of the pore in the TRPV2_RTx-ND_ structure. (**A**) S6 and pore helices of subunits A and C (left) and subunits B and D (right). Pore helices are shown in both cartoon and cylinder representation (grey). Dashed lines and values represent distances between the indicated residues. S6 helices in A and C are straight and α-helical, while the S6 in subunits B and D is bent. (**B**) Top view of the TRPV2_RTx-ND_ pore, with pore helices shown in both cartoon and cylinder representation. Dashed lines illustrate the distances between residues G604 in the selectivity filter. (**C**) Overlay of the TRPV2_RTx-ND_ pore domains (S5, S6 and pore helices). Subunit A is shown in red and subunit B in violet. The pore helix of subunit A swivels by ~10° relative to subunit B.

The pore of TRPV2_RTx-ND_ adopts a C2 symmetric arrangement (Figure 2A). The pore helices are arranged so that the carbonyl oxygens of the selectivity filter in subunits B and D line the entry to the pore while pore helices of subunits A and C tilt away from the permeation pathway. This arrangement creates a large C2 symmetric opening where the narrowest constriction between SF residues in diametrically opposing subunits A and C and B and D is ~11 Å and ~8 Å, respectively. This results in an SF with ample room to accommodate large organic cations (Figure 2B). A closer look at the pore helices reveals that this arrangement in the SF is achieved through a ~ 10° swivel of the subunit A pore helix, which brings the N-terminal part of the helix closer to S5 while distancing it from S6 (Figure 2C). The position of the pore helices controls the size and the shape of the SF, and appears to exert control over ion permeation in TRPV2. While the SF is widely open, the common gate adopts a putative intermediate conformation where two of the diagonally opposing subunits adopt a closed state, and the remaining two are open. In subunits A and C, the S6 helix adopts a straight α-helical, closed conformation, while the S6 of subunits B and D is bent and the common gate apparently open (Figure 2A). However, the overall functional state of the common gate is likely non-conductive as the gate residues from subunits A and C would presumably hinder ion permeation.

**Figure 2—figure supplement 1.**
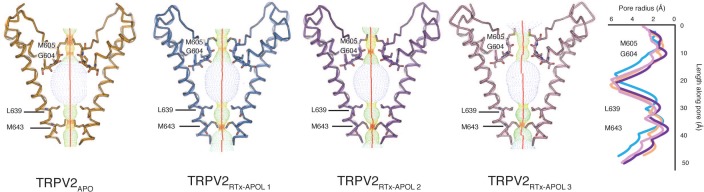
Pore comparison of TRPV2_APO_ (orange) and TRPV2_RTx-APOL 1-3_ (blue, purple and salmon, respectively). HOLE profiles (dots and graph) indicate that both the selectivity filter and the common gate are closed in TRPV2_RTx-APOL 1-3_.

In order to establish the origin of the C2 symmetry in the TRPV2_RTx-ND_ structure, we aligned subunits A and B (Cα R.M.S.D = 0.96) (Figure 3—figure supplement 5). Similar to our previous findings, this alignment shows that the two subunits diverge at the S4-S5 linker and the PH and indicates that rotation of subunits around the S4-S5_π-hinge_ appears to result in the distinct C2 symmetric arrangement observed in TRPV2_RTx-ND_ (Figure 3—figure supplements 4–5).

**Figure 3.**
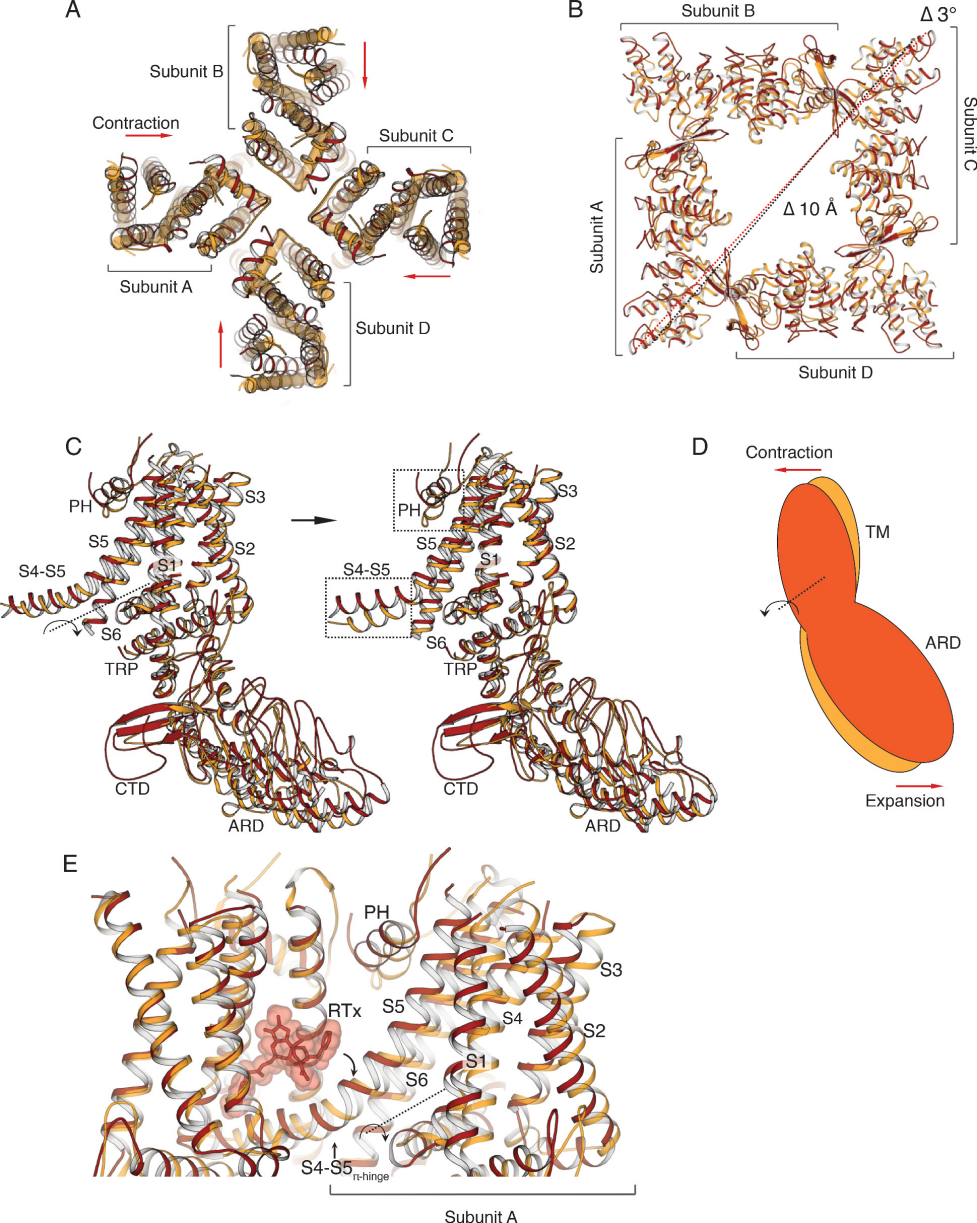
Comparison of TRPV2_RTx-ND_ (red) and TRPV2_APO_ (orange). (**A**) Overlay of TRPV2_RTx-ND_ and TRPV2_APO_, top view. TRPV2_RTx-ND_ is shown in cartoon representation and TRPV2_APO_ as cylinders. Relative to TRPV2_APO_, the TM subunits of TRPV2_RTx-ND_ exhibit contraction (red arrows). (**B**) Top view of the ARDs in TRPV2_RTx-ND_ and TRPV2_APO_. TM helices are removed for ease of viewing. Dashed lines represent distances between residues T100, showing a 10 Å expansion (Δ 10 Å) and 3° rotation (Δ 3^o^) of the TRPV2_RTx-ND_ ARD assembly relative to TRPV2_APO_. (**C**) A rigid-body rotation of TRPV2_RTx-ND_ subunit B around the S4-S5 linker achieves alignment with the subunit B from TRPV2_APO_. Following alignment, only the S4-S5 linkers and the pore helices (PH) diverge in the two subunits (dashed box). (**D**) Cartoon illustrating how the movements of the TM and the ARD in TRPV2_RTx-ND_ are coupled. The red and orange shapes represent a single subunit of TRPV2_RTx-ND_ and TRPV2_APO,_ respectively. The rotation of the subunit is manifested as ‘contraction’ in the TM domains and ‘expansion’ of the ARD. (**E**) RTx binding in the vanilloid binding pocket exerts force on the S4-S5 linker, changing the conformation of the junction from α- to π-helix, and induces the rotation of the subunit around the S4-S5_π-hinge_.

When compared to the TRPV2_APO_, the TM domains of the TRPV2_RTx-ND_ structure appear to contract in an asymmetric manner (Figure 3A), while the ARD assembly expands by ~10 Å and rotates by 3° (Figure 3B). The TM domains and the ARDs appear to move as a single rigid body, which is evident when individual subunits from TRPV2_APO_ and TRPV2_RTx-ND_ are superposed (Cα R.M.S.D = 1.9 Å) to reveal that only the S4-S5 linker and the pore helix deviate significantly in the two structures (Figure 3C). This coupled movement of the TM and ARD indicates that RTx-binding to TRPV2 in lipid membranes induces a rigid-body rotation of the entire subunit that originates at the S4-S5_π-hinge_ (Figure 3D–E).

**Figure 3—figure supplement 1.**
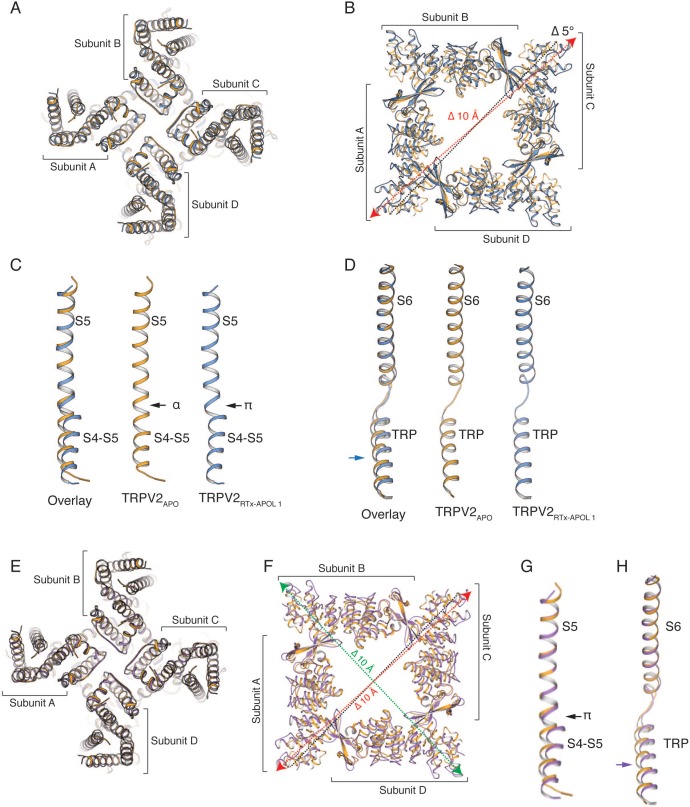
Comparison of TRPV2_RTx-APOL 1-2_ and TRPV2_APO_. (**A–D**) Comparison of TRPV2_RTx-APOL 1_ (blue) and TRPV2_APO_ (orange). (**A**), Overlay of the TM helices. Individual subunits are indicated. (**B**) Top view of the cytoplasmic domains. The TMs are removed for ease of viewing. Distance measured between residues T100 in TRPV2_APO_ (black dotted line) and TRPV2_RTx-APOL 1_ (red dotted line). The cytoplasmic assembly rotates by 5° and widens by 10 Å in the presence of RTx. (**C**) Overlay of S5 helices. In the presence of RTx, a π-helix is formed at the junction of the S4-S5 linker and the S5 helix changing the position of the S4-S5 linker. (**D**) Overlay of S6 helices and the TRP domain. The TRP domain is displaced in the presence of RTx. (**E–H**) Comparison of TRPV2_RTx-APOL 2_ (violet) and TRPV2_APO_ (orange). (**E**) Top-view of the overlay of the TM helices. Individual subunits are indicated. (**F**) Top-view of the cytoplasmic domains. The TMs are not shown for ease of viewing. Distances measured between residues T100 of diagonally opposing subunits (TRPV2_APO_ (black dotted line) and TRPV2_RTx-APOL 2_ (red and green dotted lines). (**G**) Overlay of the S5 helices shows that the S4-S5 linker adopts different conformations. The divergence originates at the π-helix. (**H**) Overlay of the S6 helices and the TRP domain shows a displacement of the TRP helix in the TRPV2_RTx-APOL 2_.

**Figure 3—figure supplement 2.**
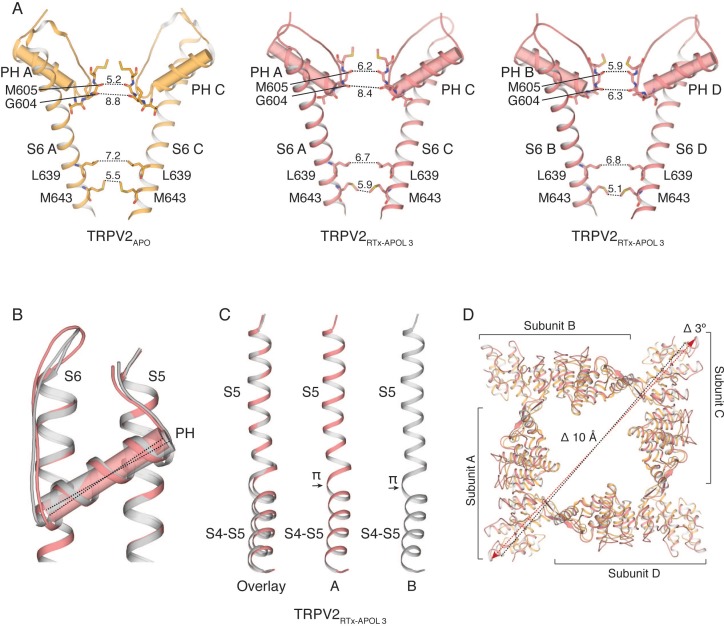
Two-fold symmetry in TRPV2_RTx-APOL 3_ (salmon). (**A**) Pore of the four-fold symmetric TRPV2_APO_ (orange) compared to the pore of the two-fold symmetric TRPV2_RTx-APOL 3_ (salmon) subunits A and C (middle) and subunits B and D (right). (**B**) Position of the pore helix in TRPV2_RTx-APOL 3_ subunit A (salmon) compared to the subunit B (grey). (**C**) Conformation of the S4-S5 linker in TRPV2_RTx-APOL 3_ subunit A (salmon) compared to subunit B (grey). (**D**) Comparison of TRPV2_RTx-APOL 3_ (salmon) and TRPV2_APO_ (orange) ARD. The TMs are removed for ease of viewing. The dashed lines represent the distance between diagonally opposite residues T100 in TRPV2_APO_ (black line) and TRPV2_RTx-APOL 3_ (red line). The ARD are rotated and expanded in TRPV2_RTx-APOL 3_.

**Figure 3—figure supplement 3.**
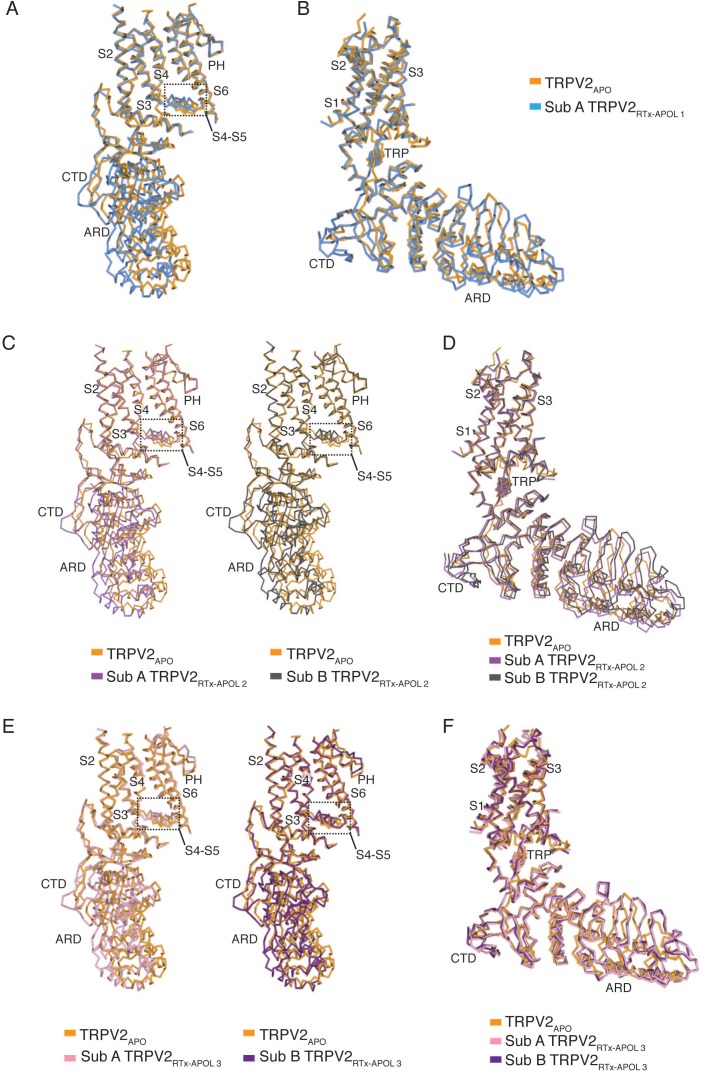
Symmetry breaking in the TRPV2_RTx-APOL 2-3_ ARD. (**A–B**) Subunit A of the four-fold symmetric TRPV2_RTx-APOL 1_ overlaid with TRPV2_APO_ shows good alignment of the TM domains, with divergence at the S4-S5 linker and the ARD. (**C**) Two-fold symmetry in the ARD and S4-S5 linker of the TRPV2_RTx-APOL 2_ structure. Subunit A (purple) overlaid with TRPV2_APO_ (orange) (left). Subunit B (dark grey) overlaid with TRPV2_APO_ (orange) (right). In both subunits, TM domains are aligned but ARD and the S4-S5 linker (dashed line box) diverge. (**D**) TRPV2_RTx-APOL 2_ subunits A and B (dark grey) assume distinct conformations in the ARD. (**E**), Two-fold symmetry in the ARD and S4-S5 linker of the TRPV2_RTx-APOL 3_ structure. Subunit A (salmon) overlaid with TRPV2_APO_ (orange) (left). Subunit B (purple) overlaid with TRPV2_APO_ (orange) (right). Similar to TRPV2_RTx-APOL 2_, TM domains are aligned but ARD and the S4-S5 linker (dashed line box) diverge. (**D**) TRPV2_RTx-APOL 3_ subunits A and B (purple) assume distinct conformations in the ARD, albeit to lesser extent than TRPV2_RTx-APOL 2_.

**Figure 3—figure supplement 4.**
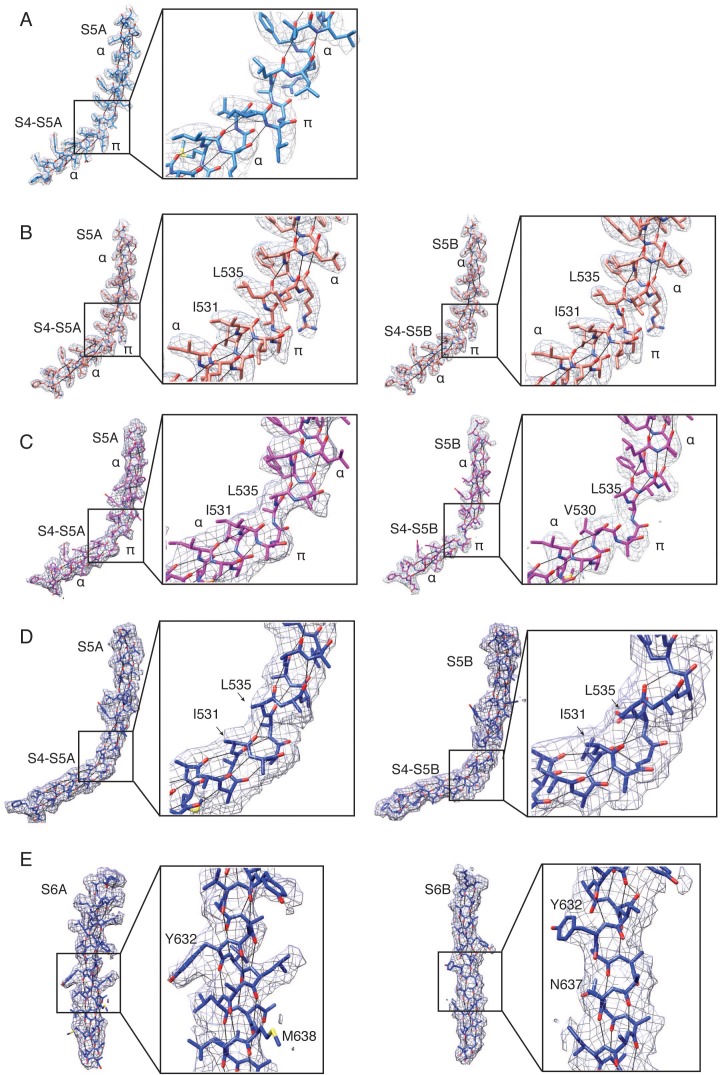
Electron density around the S4-S5 linker π-helices in TRPV2_RTx-APOL 1_ (**A**), TRPV2_RTx-APOL 2_ (**B**) and TRPV2_RTx-APOL 3_ (**C**). (**A**) TRPV2_RTx-APOL 1_ density is contoured at level 0.06 and radius 2. Only subunit A is shown. The inset shows a close-up of the junction between the S4-S5 linker and the S5 helix. (**B**) TRPV2_RTx-APOL 2_ density is contoured at level 0.04 and radius 2. Subunits A and B are shown. The insets show a close-up of the junction between the S4-S5 linker and the S5 helix. (**C**) TRPV2_RTx-APOL 1_ density is contoured at level 0.015 and radius 2. Subunits A and B are shown. The insets show a close-up of the junction between the S4-S5 linker and the S5 helix. (**D–E**) Electron density in TRPV2_RTx-ND_ contoured at level 0.01. (**D**) Density around the S5 helix and the S4-S5 linker in TRPV2_RTx-ND_ subunits A and B. The insets show a close-up of the junction between the S4-S5 linker and the S5 helix. (**E**) Density around TRPV2_RTx-ND_ helices S6A and S6B. The inset shows a close-up of the hydrogen bonds in the helix.

**Figure 3—figure supplement 5.**
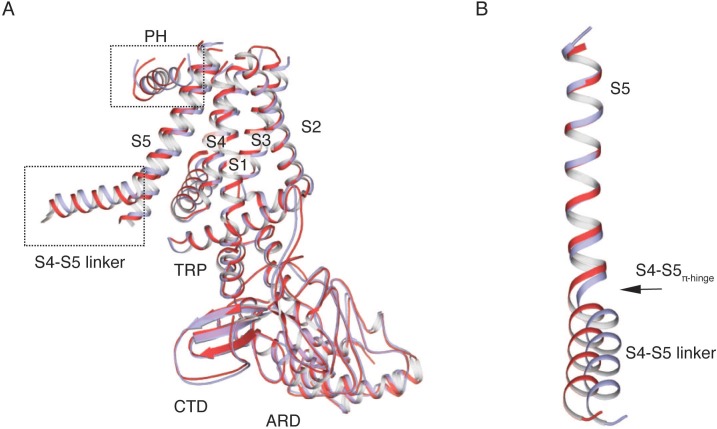
Comparison of TRPV2_RTx-ND_ subunits A (red) and B (violet). (**A**) Overlay of the subunits. The regions that diverge from the overlay, the S4-S5 linker and the pore helix (PH), are indicated by a dashed line box. (**B**) Overlay of S5 helices. The alignment diverges at the S4-S5 linker π-helix (S4-S5_π-hinge_) giving rise to different conformations of the S4-S5 linker in the two subunits.

**Figure 4.**
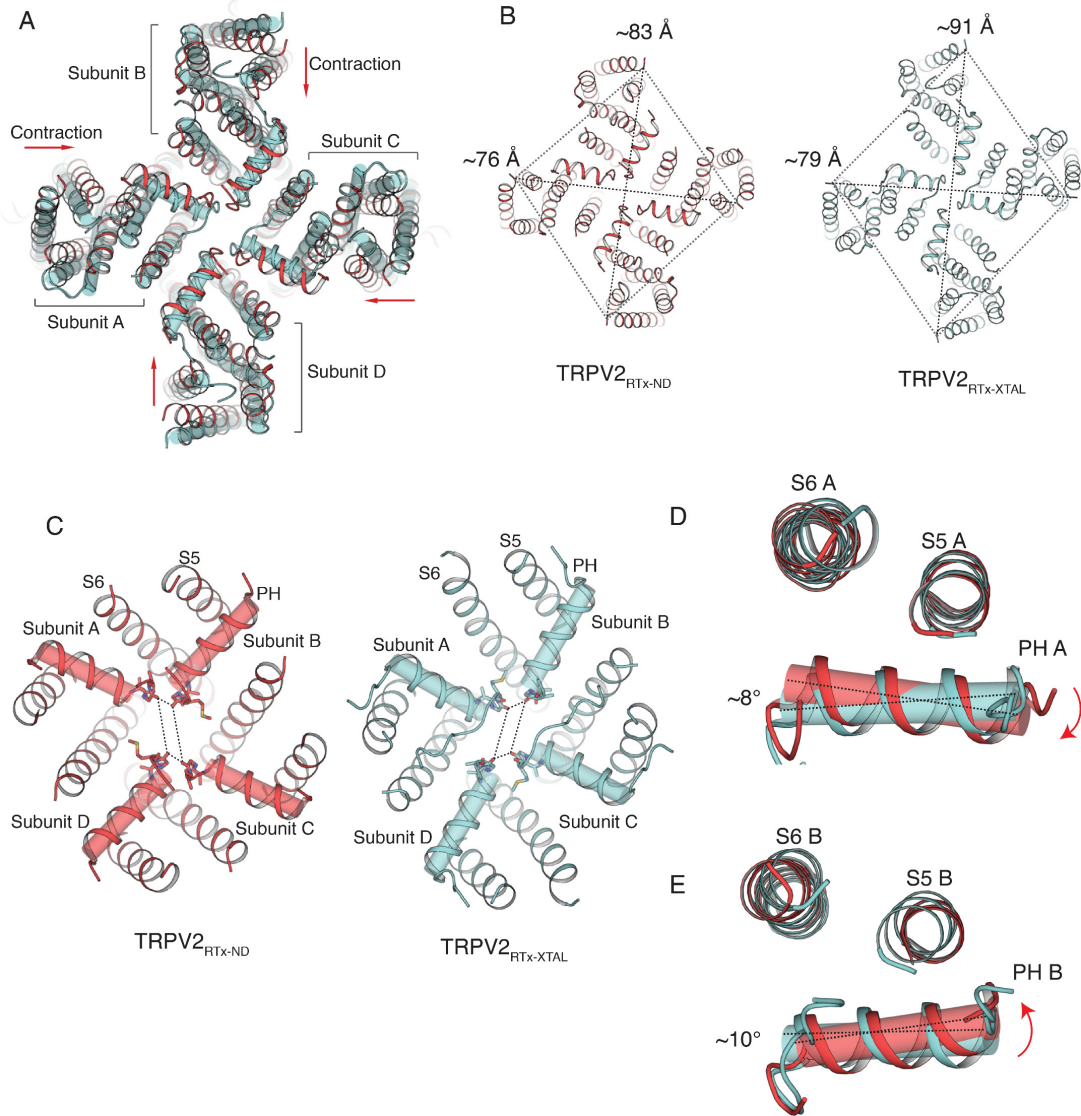
Comparison of TRPV2_RTx-ND_ (red) and TRPV2_RTx-XTAL_ (cyan). (**A**) Overlay of TRPV2_RTx-ND_ and TRPV2_RTx-XTAL_, top view. TRPV2_RTx-ND_ is shown in cartoon representation and TRPV2_RTx-XTAL_ as cylinders. Relative to TRPV2_RTx-XTAL_, the TM domains of TRPV2_RTx-ND_ are contracted (red arrows). (**B**) Comparison of two-fold symmetry in TRPV2_RTx-ND_ and TRPV2_RTx-XTAL_. Dashed lines represent distances between residues A427. The distances between diagonally opposing subunits are indicated. (**C**) Top view of the SF in TRPV2_RTx-ND_ and TRPV2_RTx-XTAL_. Pore helices are shown in both cartoon and cylinder representation. Dashed lines represent distances between residues G604 in the selectivity filter. (**D–E**) Overlay of the pore domains of TRPV2_RTx-ND_ and TRPV2_RTx-XTAL_ subunit A (**D**) and subunit B (**E**) shows that the pore helices A and B in TRPV2_RTx-ND_ swivel by ~8° and ~10°, respectively, compared to TRPV2_RTx-XTAL_.

Interestingly, the TRPV2_RTx-ND_ structure exhibits different degrees of reduced symmetry from the previously determined crystal structure of TRPV2 in complex with RTx (TRPV2_RTx-XTAL_) (Zubcevic et al., 2018a). Compared to the TRPV2_RTx-XTAL_, the TM domains of TRPV2_RTx-ND_ contract in an two-fold symmetric manner (Figure 4A). This conformational change, which stems from rotation of individual TRPV2_RTx-ND_ subunits around the S4-S5_π-hinge_ (Figure 4—figure supplement 1), results in an overall fold that is closer to C4 symmetry than that of the TRPV2_RTx-XTAL_ (Figure 4B). However, while the TRPV2_RTx-ND_ helices S1-S6 adopt a more C4 symmetric arrangement, the pore helices and the SF remain distinctly C2 symmetric (Figure 4C). Remarkably, the SF of TRPV2_RTx-ND_ is wider than that of TRPV2_RTx-XTAL,_ and the two structures display different C2 symmetric openings at the SF (Figure 4C). The two different conformations result from both the different arrangements of subunits and changes in the position and tilt angle of the pore helices (Figure 4D–E). In the TRPV2_RTx-XTAL_ structure, the pore helices of subunits B and D, which assume a widened conformation, are free of interactions with the pore domain, while a network of interactions (presumably hydrogen bonds) between Y542-T602-Y627 in subunits A and C tethers the pore helices to S5 and S6. Our previous work showed that disruption of these interactions is detrimental to the permeation of large organic cations but has no effect on permeation of metal ions (Zubcevic et al., 2018a). Interestingly, the putative hydrogen bond triad is disrupted in all four subunits of the TRPV2_RTx-ND_ structure (Figure 4—figure supplement 2). Nevertheless, the SF assumes a fully open state that can potentially accommodate passage of a large cation. This suggests that the putative hydrogen bond triad, while not a feature of the fully open SF, is an essential part of the transition between closed and open states of the channel.

**Figure 4—figure supplement 1.**
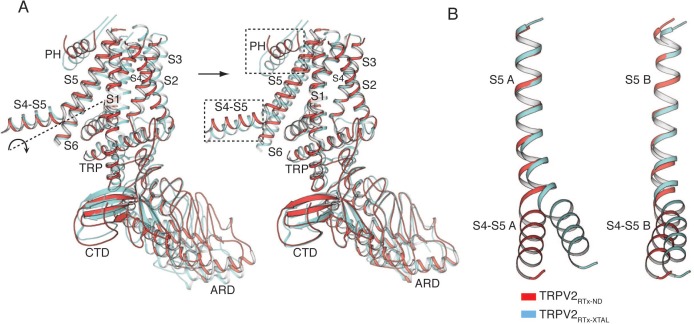
Comparison of subunits B in TRPV2_RTx-ND_ (red) and TRPV2_RTx-XTAL_ (cyan). (**A**) Rotation of subunit B from TRPV2_RTx-ND_ around the S4-S5_π-hinge_ aligns it to subunit B from TRPV2_RTx-XTAL._ The S4-S5 linker and the PH (dashed box) diverge from the alignment. Rotation axis indicated with dashed line and arrow. (**B**) Overlay of TRPV2_RTx-ND_ and TRPV2_RTx-XTAL_ S5 helices from subunit A (left) and subunit B (right) show that the S4-S5 linkers assume different conformations.

**Figure 4—figure supplement 2.**
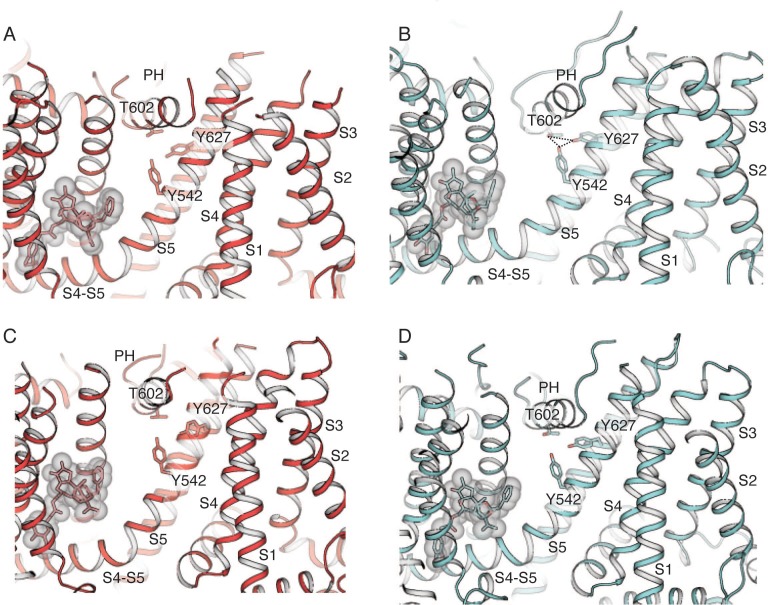
Interactions between the pore helix (PH) and S5 and S6. RTx is shown in stick and transparent surface representations. (**A–B**) Side view of subunits A in TRPV2_RTx-ND_(A) and TRPV2_RTx-XTAL_(B). The putative hydrogen bond triad (Y542-T602-Y627) is present in subunit A of TRPV2_RTx-XTAL_. (**C–D**) The triad is broken in TRPV2_RTx-ND_. Side view of subunits B in TRPV2_RTx-ND_(C) and TRPV2_RTx-XTAL_(D). The interaction network is absent in both structures.

**Figure 4—figure supplement 3.**
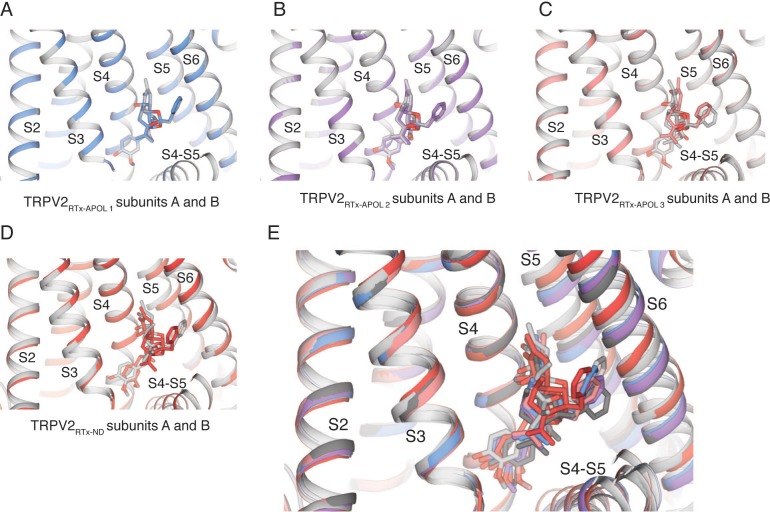
Binding of RTx in TRPV2_RTx-APOL_ and TRPV2_RTx-ND_. (**A**) Binding of RTx in subunit A (blue) and B (grey) of the C4 symmetric TRPV2_RTx-APOL 1_. (**B**) Binding of RTx in subunit A (violet) and B (grey) of the C2 symmetric TRPV2_RTx-APOL 2_. (**C**) Binding of RTx in subunit A (salmon) and B (grey) of the C2 symmetric TRPV2_RTx-APOL 3_. (**D**) Binding of RTx in subunit A (red) and B (grey) of the C2 symmetric TRPV2_RTx-ND_. (**E**) Overlay of TRPV2_RTx-APOL 1_ subunit A (blue), TRPV2_RTx-APOL 2_ subunit A (violet), TRPV2_RTx-APOL 3_ subunit A (salmon) and subunit B (dark grey), TRPV2_RTx-ND_ subunit A (red) and subunit B (light grey) shows the different binding poses of RTx captured in this study.

**Figure 4—figure supplement 4.**
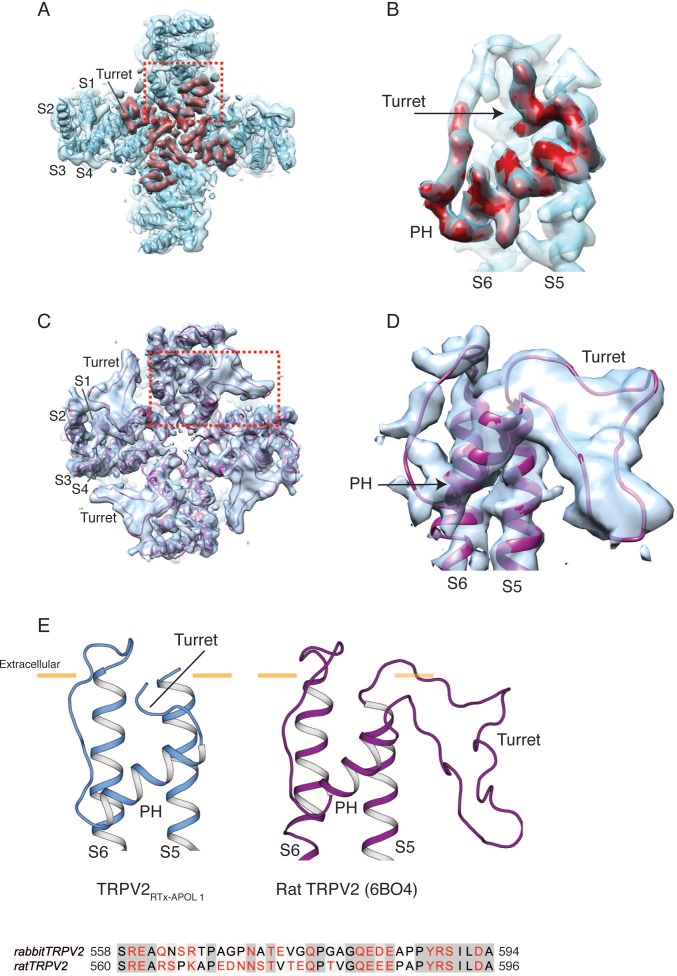
The pore turret in TRPV2_RTx-APOL 1_ (blue) and rat TRPV2 (PDB 6BO4, purple). (**A**) Top view of the map and model of TRPV2_RTx-APOL 1_ with the pore domain indicated by dashed red box. The density containing S5, S6, the PH, pore loop and turret is colored in red. (**B**) Side view of the map and model of the pore domain in TRPV2_RTx-APOL 1_. The density containing S5, S6, the PH, pore loop and turret is colored in red. S5, S6, PH and pore turret are indicated. (**C**) Top view of the map and model of rat TRPV2 with the pore domain indicated by dashed red box. (**D**) Side view of the map and model of the pore domain in rat TRPV2. S5, S6, PH and pore turret are indicated. (**E**) Position of the turret relative to the membrane (yellow lines) in TRPV2_RTx-APOL 1_ and rat TRPV2. The sequence of the turret shows conservation (grey boxes) and amino acids colored in red indicate charged or polar residues.

**Figure 4—figure supplement 5.**
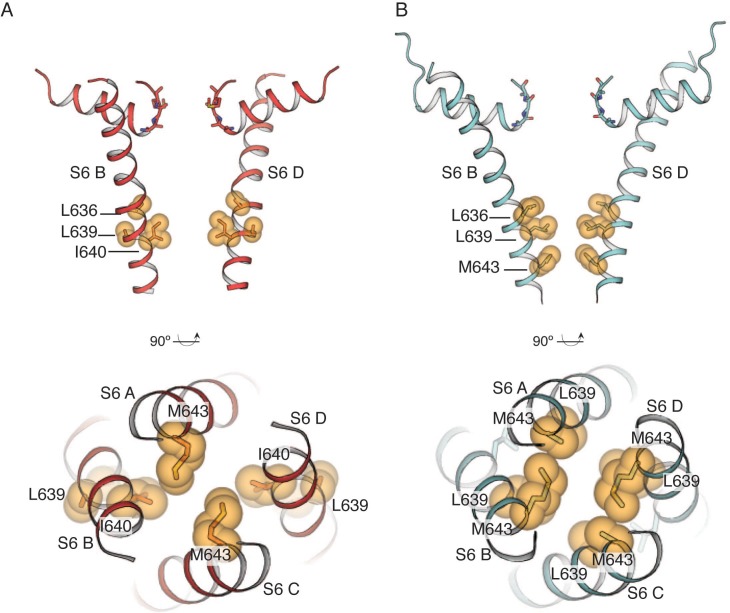
The common gate in TRPV2_RTx-ND_ (red) and TRPV2_RTx-XTAL_ (cyan). (**A**) Side view of the TRPV2_RTx-ND_ pore showing subunits B and D (top). Gate residue I640 is shown in yellow spheres, along with the hydrophobic residues L636 and L639 (side chains not built). Bottom-up view (bottom) shows the contribution of all four subunits to the common gate (M643 in subunits A and C, I640 in subunits B and D). (**B**) Side view of the TRPV2_RTx-XTAL_ pore, showing subunits B and D (top). Gate residue M643 is shown in yellow spheres, along with hydrophobic pore lining residues L636 and L639. Bottom-up view of the common gate (bottom) shows gate residues M643 (side chain not built in subunits A and C).

**Figure 4—figure supplement 6.**
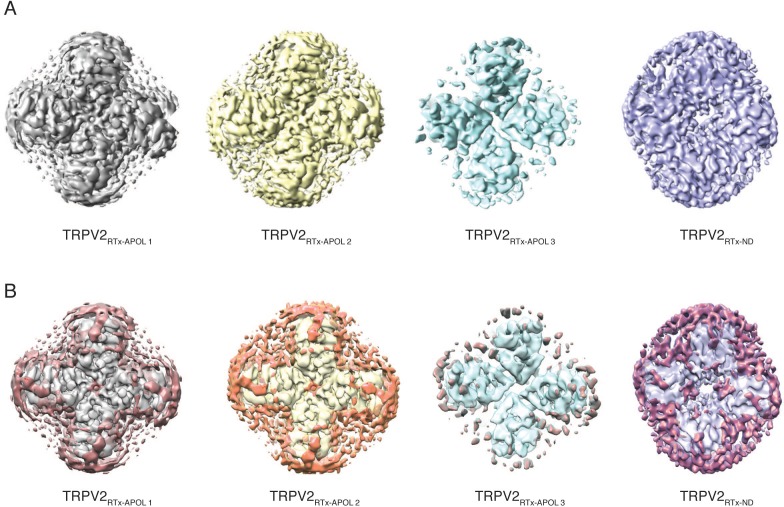
The amphipol and nanodisc clouds. (**A**) Top-view of the TRPV2_RTx-APOL 1-3_ and TRPV2_RTx-ND_ maps. (**B**) Top-view of the TRPV2_RTx-APOL 1-3_ and TRPV2_RTx-ND_ maps with the non-protein density colored in red. All maps were filtered to 5 Å and contoured at level 0.015.

It is interesting to point out that extensive rearrangements around the SF and the PH during gating have thus far only been observed in structural studies of TRPV1 (Cao et al., 2013b) and TRPV2 (Zubcevic et al., 2018a) channels. In the non-conductive state, the SFs of the remaining members of the TRPV subfamily (TRPV3-TRPV6 [Zubcevic et al., 2018b; Singh et al., 2018; Deng et al., 2018; Hughes et al., 2018; McGoldrick et al., 2018]) adopt a conformation that is wide enough to accommodate a semi-hydrated cation, and do not move appreciably during channel activation. This may indicate that TRPV1 and TRPV2 are the only members of the TRPV subfamily that possess a gate at the SF, and that the coupling of structural elements necessary for activation of these channels differs from that of TRPV3-TRPV6 (Zhang et al., 2019).

As observed in our previous study (Zubcevic et al., 2018a), RTx assumes different binding poses in subunits of the C2-symmetric structures, both in the amphipol and the nanodisc samples (Figure 4—figure supplement 3) which may lead to the distinct conformations observed in these channels.

Despite the use of a full-length rabbit TRPV2 construct in this study, we were not able to confidently resolve the entire loop connecting S5 to the pore helix known as the ‘pore turret’. Interestingly, a recent structure of rat TRPV2 with the pore turret resolved showed that this region, which contains a large number of charged and polar residues, occupies the space within the membrane plane between S5 and the Voltage Sensor-Like Domain (VSLD) (Dosey et al., 2019). While the density in our cryo-EM maps was not of sufficient quality to build the entire pore turret with confidence, we do observe density following the S5 helix and preceding the pore helix. However, the direction of this density is perpendicular to the membrane and does not agree with the structure reported for rat TRPV2 (Figure 4—figure supplement 4). Indeed, the pore turret is the least conserved region amongst the TRPV2 orthologs, and the variations in its sequence might indicate that the turret adopts different conformations in TRPV2 channels from different species. Nevertheless, our study clearly shows that the omission of this region from the construct used in the crystallographic study of the TRPV2/RTx complex is not the cause of the C2 symmetry.

While both TRPV2_RTx-ND_ and TRPV2_RTx-XTAL_ structures adopt C2 symmetry, the distinct arrangement of subunits within the two channels suggests that the structures represent different functional states. We propose that TRPV2_RTx-XTAL_ precedes TRPV2_RTx-ND_ in the conformational activation trajectory based on two observations. Firstly, the common gate is fully closed in the TRPV2_RTx-XTAL_ while it adopts an apparently partially open conformation in TRPV2_RTx-ND_ (Figure 4—figure supplement 5). Secondly, our previous studies have shown that the putative hydrogen bond network between S5 and S6 and the pore helix is essential for the channel’s ability to transition to a fully open SF that can accommodate large organic cations (Zubcevic et al., 2018a). Nevertheless, in TRPV2_RTx-ND_ the pore helices do not interact with S5 and S6 and the SF is fully open. We therefore propose that the conformational step that requires the presence of the putative hydrogen bond triad precedes the open SF conformation seen in TRPV2_RTx-ND_.

## Discussion

Here, we have conducted a study that reveals symmetry transitions associated with gating of the TRPV2 channel by RTx. Interestingly, our data shows that RTx induces C2 symmetric conformations of TRPV2 in both amphipol and nanodiscs, and it thereby negates the hypothetical role of crystallization artefacts and crystal packing bias in stabilising two-fold symmetry. Similarly, C2 symmetry in TRPV2 is independent of the presence or absence of the pore turret region, suggesting that this region does not play an essential role in the regulation of the SF in rabbit TRPV2. Our study, similar to a previously published study of the magnesium channel CorA (Matthies et al., 2016), also emphasizes the notion that careful inspection of the intermediate maps and conservative application of symmetry during refinement of cryo-EM data can result in valuable insights into gating transitions and intermediate states. In addition, we have also investigated how amphipols and nanodiscs affect the conformational space that can be accessed during ligand gating of TRPV2.

While both TRPV2_RTx-APOL_ and TRPV2_RTx-ND_ are C2 symmetric, the two-fold symmetry in TRPV2_RTx-APOL_ is confined to regions that are not bound by the amphipol polymer. This is evident in the fact that the TM domains, which are in contact with the amphipol, largely retain four-fold symmetry and the common gate and the SF remain firmly closed, while the ARD exhibit symmetry breaking, rotation and lateral expansion. These data, while adding valuable data points to the conformational landscape of TRPV2, also illustrate the potential caveats of using amphipols in studies of conformational changes in the transmembrane domains of proteins, as they appear to constrict the TM domains and stabilize low-energy pre-open states (Figure 4—figure supplement 6). However, at this time we caution against any generalized conclusions about the effect of amphipols and look forward to more systematic studies that will address these issues in the future. The TRPV2_RTx-ND_ dataset yielded a single, two-fold symmetric structure thus giving strong evidence that RTx stabilizes two-fold symmetric conformational states in the TRPV2 channel in lipid membranes. The ARDs in the TRPV2_RTx-ND_ structure echo the conformational changes observed in TRPV2_RTx-APOL._ However, in nanodiscs TRPV2 is captured with its SF fully open and its common gate in an intermediate conformation where the gate is apparently open in one set of diagonally opposing subunits and closed in the other. In this structure, the opening of the SF occurs according to a mechanism previously observed in the crystallographic study of the TRPV2/RTx complex where RTx binding in the vanilloid pocket, above the S4-S5_π-hinge_, induces a rigid body rotation of the entire subunit. In turn, the rotation causes a break in the interaction network between the pore helix and helices S5 and S6, allowing the pore helices to reposition and the SF to open (Zubcevic et al., 2018a).

**Figure 5.**
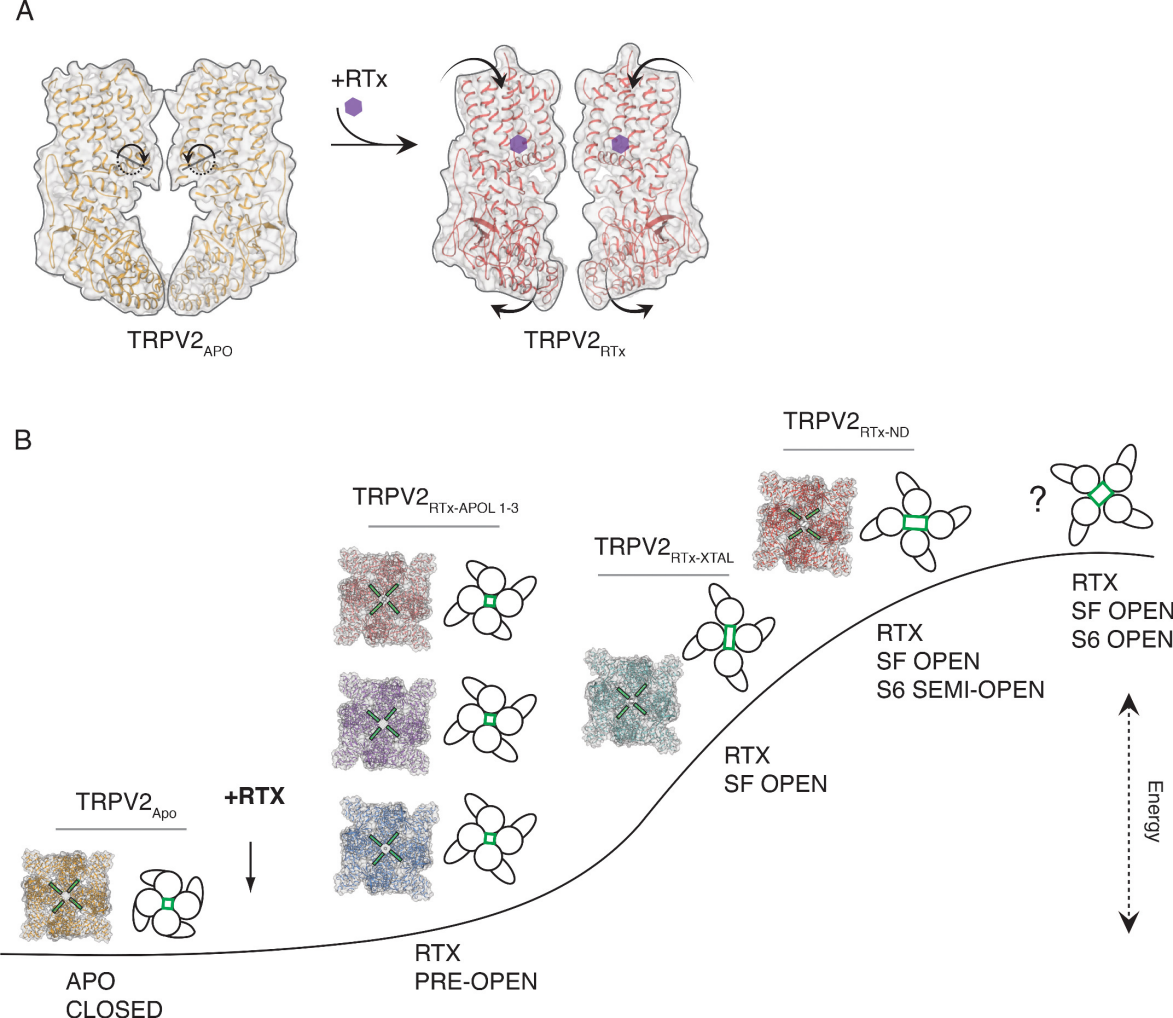
Conformational states associated with RTx-mediated gating of TRPV2. (**A**) TRPV2 subunit rotation upon binding of RTx. Rotation axis and direction are indicated in dashed line and circular arrow in apo TRPV2 (left). The rotation results in contraction of the TM domains and widening of the cytoplasmic assembly (right). (**B**) Hypothetical trajectory of TRPV2 gating with associated conformational states. Upon addition of RTx, TRPV2 first enters low-energy pre-open states that are characterized by rotation, widening and symmetry breaking in the ARD (TRPV2_RTx-APOL 1-3,_ models shown in cartoon and surface representation). In the next step, the channel assumes C2 symmetric state with an open SF, but closed common (S6) gate (TRPV2_RTx-XTAL,_ model shown in cartoon and surface representation). This is followed by a less C2 symmetric state with an open SF and semi-open common (S6) gate (TRPV2_RTx-ND,_ model shown in cartoon and surface representation). Finally, we propose that the channel assumes a high-energy fully open state that is C4 symmetric but might have C2 symmetry in the SF. The SF is indicated in green in models and cartoons.

Interestingly, however, the TRPV2_RTx-ND_ structure differs from the previously obtained TRPV2_RTx-XTAL_. While both structures assume C2 symmetric conformations, the TRPV2_RTx-ND_ channel appears to make a return toward C4 symmetry. Because the SF in TRPV2_RTx-ND_ is fully open, and the common gate adopts an apparently partially open conformation, we reason that TRPV2_RTx-ND_ follows the TRPV2_RTx-XTAL_ structure in the conformational trajectory of the channel. Therefore, it is possible that TRPV2, as it travels toward the final open state where both the SF and the common gate are fully open, would adopt further conformations that increasingly approximate C4 symmetry (Figure 5). However, it is interesting to note that while the overall fold of TRPV2_RTx-ND_ indeed is more C4 symmetric than that of TRPV2_RTx-XTAL_, the extent of C2 symmetry is not diminished in its SF. Because the symmetry of the SF does not appear to be dictated by the symmetry of the overall channel, we cannot exclude the possibility that the final open state might indeed possess a C2 symmetric SF while otherwise adopting a nearly C4 symmetric conformation. Our previous functional studies have suggested that C2 symmetric states play a role in the permeation of large organic cations and consequently in the full opening of the SF (Zubcevic et al., 2018a). Hence, the channel might be utilizing C2 symmetric states as means to achieve full opening in a step-wise manner. Similar C2 symmetric states elicited by ligand binding have been observed in TRPV3 (Zubcevic et al., 2018b) and TRPM2 (Yin et al., 2018) channels, which opens up the possibility that C2 symmetry might be widely associated with gating in members of the TRP channel superfamily. Intriguingly, a recent cryo-EM study of the human BK channel reconstituted in liposomes showed that this channel also enters C2 symmetric states (Tonggu and Wang, 2018), suggesting that two-fold symmetry might also play a role in the molecular mechanisms of other tetrameric ion channels.

**Figure 6.**
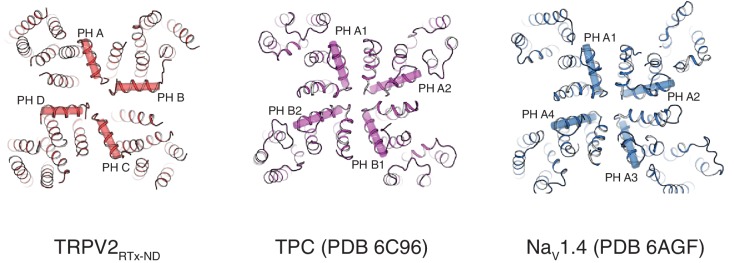
Comparison of TRPV2_RTx-ND_ (red), TPC1 (PDB 6C96, purple) and Na_V_1.4 (PDB 6AGF, blue). Top view, pore helices are indicated.

Two-fold symmetry is a well-stablished feature of mammalian Na^+^ selective Two Pore Channels (TPCs) and Voltage Gated Sodium channels (Na_V_) (She et al., 2018; Shen et al., 2017; Pan et al., 2018; Shen et al., 2018). Interestingly, the arrangement of pore helices in TRPV2_RTx-ND_ resembles that observed in TPC and Na_V_ (Figure 6) and the selectivity filters in all three channels form a 'coin-slot' (Hille, 1971) opening. However, while the selectivity filters of TPC and Na_V_ remain static during channel gating in order to maintain the structure necessary for Na^+^ selectivity, the SF of TRPV2 displays a large degree of plasticity. Moreover, the two-fold symmetry observed in TRPV2 is unique in that it arises in response to conformational changes in the TM domains induced by ligand binding. By contrast, the two-fold symmetry in TPC and Na_V_ stems from the arrangement of their respective homologous tandem repeats.

## Materials and methods

**Key resources table:** 

Reagent type (species) or resource	Designation	Source or reference	Identifiers	Additional information
Cell line	DH10Bac *E. coli*	ThermoFisher Scientific	10361012	
Cell line	Sf9	ATCC	CRL-1711	RRID:CVCL_0549
Recombinant DNA reagent	rabbit TRPV2	Genscript	Pubmed Accession No. XM_017349044	
Recombinant DNA reagent	Bac-to-Bac Baculovirus Expression System	ThermoFisher Scientific	10359016	
Recombinant DNA reagent	MSP2N2 scaffold protein	Stephen Sligar laboratory	Addgene:Cat#29520	PMID:20817758
Chemical compound, drug	*n*-dodecyl-β-d- maltopyranoside(DDM)	Anatrace	D310	
Chemical compound, drug	Cholesteryl Hemisuccinate	Anatrace	CH210	
Chemical compound, drug	Amphipol A8-35	Anatrace	A835	
Chemical compound, drug	TRIS	Fisher Scientific	BP152	
Chemical compound, drug	NaCl	Fisher Scientific	S271	
Chemical compound, drug	CaCl2	Fisher Scientific	C70	
Chemical compound, drug	leupeptin	GoldBio	L-010	
Chemical compound, drug	pepstatin	GoldBio	P-020	
Chemical compound, drug	aprotinin	GoldBio	A-655	
Chemical compound, drug	DNase I	GoldBio	D-301	
Chemical compound, drug	β-mercapto ethanol	Sigma Aldrich	M3148	
Chemical compound, drug	PMSF	Sigma Aldrich	P7626	
Chemical compound, drug	anti-FLAG resin	Sigma Aldrich	A4596	
Chemical compound, drug	Resiniferatoxin	Sigma Aldrich	R8756	
Chemical compound, drug	Bio-Beads SM-2	BioRad	152–8920	
Chemical compound, drug	1,2-dimyristoyl- *sn*-glycero-3- phosphocholine	Avanti Polar Lipids	850345P	
Chemical compound, drug	1-palmitoyl-2- oleoyl-*sn*-glycero-3- phosphocholine (POPC)	Avanti Polar Lipids	850457C	
Chemical compound, drug	1-palmitoyl-2-oleoyl -*sn*-glycero-3- phosphoethanolamine (POPE)	Avanti Polar Lipids	850757C	
Chemical compound, drug	1-palmitoyl-2-oleoyl- *sn*-glycero-3-phospho- (1'-*rac*-glycerol) (POPG)	Avanti Polar Lipids	840457C	
Other	Whatman No. one filter paper	Sigma Aldrich	WHA1001325	
Other	UltrAuFoil R1.2/1.3 300-mesh grid	Electron Microscopy Sciences	Q350AR13A	
Software, algorithm	MotionCor2	Zheng et al. (2017)	http://msg.ucsf.edu/em/software/motioncor2.html	RRID:SCR_016499
Software, algorithm	GCTF	Zhang (2016)	https://www.mrc-lmb.cam.ac.uk/kzhang/	RRID:SCR_016500
Software, algorithm	RELION 3.0	Zivanov et al. (2018)	https://www2.mrc-lmb.cam.ac.uk/relion/	RRID:SCR_016274
Software, algorithm	Coot	Emsley and Cowtan (2004)	https://www2.mrc-lmb.cam.ac.uk/personal/pemsley/coot/	RRID:SCR_014222
Software, algorithm	Phenix	Adams et al. (2010)	http://phenix-online.org/	RRID:SCR_014224
Software, algorithm	Molprobity	Chen et al. (2010)	http://molprobity.biochem.duke.edu/index.php	RRID:SCR_014226
Software, algorithm	UCSF Chimera	Pettersen et al. (2004)	https://www.cgl.ucsf.edu/chimera/	RRID:SCR_004097
Software, algorithm	Pymol	Shrödinger LLC	https://pymol.org/2/	RRID:SCR_000305
Other	Cryo-electron microscopy structure of rabbit TRPV2 ion channel	Zubcevic et al. (2018b)	PDB ID 5AN8	PMID:26779611
Other	Cryo-electron microscopy structure of rabbit TRPV2 ion channel	Zubcevic et al. (2018a)	EMDB ID EMD-6455	PMID:26779611
Other	Crystal structure of the TRPV2 ion channel in complex with RTx	Zubcevic et al. (2018a)	PDB ID 6BWJ	PMID:29728656

### Protein expression and purification

The construct for the RTx sensitive, full-length rabbit TRPV2 (TRPV2_RTx_) was prepared by introducing four point mutations (F470S, L505M, L508T and Q528E) into the synthesized full-length rabbit TRPV2 gene (Zhang et al., 2016). The construct was cloned into a pFastBac vector with a C-terminal FLAG affinity tag and used for baculovirus production according to manufacturers’ protocol (Invitrogen, Bac-to-Bac). The protein was expressed by infecting Sf9 cells with baculovirus at a density of 1.3 M cells ml^−1^ and incubating at 27^o^C for 72 hr in an orbital shaker. Cell pellets were collected after 72 hr and resuspended in buffer A (50 mM TRIS pH8, 150 mM NaCl, 2 mM CaCl_2_, 1 μg ml^−1^ leupeptin, 1.5 μg ml^−1^ pepstatin, 0.84 μg ml^−1^ aprotinin, 0.3 mM PMSF, 14.3 mM β-mercaptoethanol, and DNAse I) and broken by sonication (3 × 30 pulses).

For the amphipol-reconstituted TRPV2 (TRPV2_RTx-APOL_) sample, the lysate was supplemented with 40 mM Dodecyl β-maltoside (DDM, Anatrace), 4 mM Cholesteryl Hemisuccinate (CHS, Anatrace) and 2 μM RTx and incubated at 4^o^C for 1 hr. Insoluble material was removed by centrifugation (8000 g, 30 min), and anti-FLAG resin was added to the supernatant for 1 hr at 4^o^C.

After binding, the anti-FLAG resin was loaded onto a Bio-Rad column and a wash was performed with 10 column volumes of buffer B (50 mM TRIS pH8, 150 mM NaCl, 2 mM CaCl_2_, 1 mM DDM, 0.1 mM CHS, 0.1 mg ml^−1^ 1,2-dimyristoyl-*sn*-glycero-3-phosphocholine (DMPC, Avanti Polar Lipids), 2 μM RTx) before elution in five column volumes of buffer C (50 mM TRIS pH8, 150 mM NaCl, 2 mM CaCl_2_, 1 mM DDM, 0.1 mM CHS, 0.1 mg ml^−1^ DMPC, 2 μM RTx, 0.1 mg ml^−1^ FLAG peptide).

The eluate was concentrated and further purified by gelfiltration on a Superose six column. The peak fractions were collected, mixed with Amphipol A8-35 (Anatrace) in a 1:10 ratio and incubated for 4 hr at 4^o^C. Subsequently, Bio-Beads SM-2 (Bio-Rad) were added to a 50 mg ml^−1^ concentration and incubated at 4^o^C overnight to remove detergent.

After reconstitution, the protein was subjected to a second round of gelfiltration on a Superose six column in buffer D (50 mM TRIS pH8, 150 mM NaCl, 2 μM RTx), the peak fractions were collected and concentrated to 2–2.5 mg ml^−1^ for cryo-EM.

For the nanodisc reconstituted TRPV2 (TRPV2_RTx-ND_), the lysate was supplemented with 40 mM Dodecyl β-maltoside (DDM, Anatrace) and 2 μM RTx and incubated at 4^o^ C for 1 hr. The solution was cleared by centrifugation (8000 g, 30 min), and anti-FLAG resin was added to the supernatant for 1 hr at 4^o^C.

After binding, the anti-FLAG resin was loaded onto a Bio-Rad column and a wash was performed with 10 column volumes of buffer B_noCHS_ (50 mM TRIS pH8, 150 mM NaCl, 2 mM CaCl_2_, 1 mM DDM, 0.1 mg ml^−1^ DMPC, 2 μM RTx) before elution in five column volumes of buffer C_noCHS_ (50 mM TRIS pH8, 150 mM NaCl, 2 mM CaCl_2_, 1 mM DDM, 0.1 mg ml^−1^ DMPC, 2 μM RTx, 0.1 mg ml^−1^ FLAG peptide).

The eluate from the anti-FLAG resin was concentrated to ~500 μl. A 10 mg ml^−1^ 3:1:1 mixture of lipids 1-palmitoyl-2-oleoyl-*sn*-glycero-3-phosphocholine (POPC), 1-palmitoyl-2-oleoyl-*sn*-glycero-3-phosphoethanolamine (POPE), 1-palmitoyl-2-oleoyl-*sn*-glycero-3-phospho-(1'-*rac*-glycerol) (POPG) was dried under argon, resuspended in 1 ml 50 mM Tris pH8, 150 mM NaCl and clarified by extrusion, before being incubated for 1 hr with 10 mM DDM. The membrane scaffold protein MSP2N2 was prepared as previously described (Ritchie et al., 2009). The concentrated TRPV2 was combined with MSP2N2 and the prepared lipid mixture in a 1:3:200 ratio and incubated at 4^o^C for 1 hr. After the initial incubation, 50 mg ml^−1^ Bio-Beads SM-2 were added and the mixture was incubated for another hour at 4^o^C, following which the reconstitution mixture was transferred to a fresh batch of Bio-Beads SM-2 at 50 mg ml^−1^ and incubated overnight at 4^o^C. Finally, the reconstituted channels were subjected to gelfiltration on Superose six in buffer D, the peak fractions collected and concentrated to 2–2.5 mg ml^−1^ for cryo-EM.

### Cryo-EM sample preparation

TRPV2_RTx-APOL_ and TRPV2_RTx-ND_ were frozen using the same protocol. Before freezing, the concentrated protein sample was supplemented with 300 μM RTx and incubated ~30 min at 4°C. 3 μl sample was dispensed on a freshly glow discharged (30 s) UltrAuFoil R1.2/1.3 300-mesh grid (Electron Microscopy Sciences), blotted for 3 s with Whatman No. one filter paper using the Leica EM GP2 Automatic Plunge Freezer at 23°C and >85% humidity and plunge-frozen in liquid ethane cooled by liquid nitrogen.

### Cryo-EM data collection

Data for both TRPV2_RTx-APOL_ and TRPV2_RTx-ND_ were collected using the Titan Krios transmission electron microscope (TEM) operating at 300 keV using a Falcon III Direct Electron Detector operating in counting mode at a nominal magnification of 75,000x corresponding to a physical pixel size of 1.08 Å/pixel.

For the TRPV2_RTx-APOL_ 1293 movies (30 frames/movie) were collected using a 60 s exposure with an exposure rate of ~0.8 e^-^/pixel/s, resulting in a total exposure of 42 e^-^/Å (Cao et al., 2013a) and a nominal defocus range from −1.25 µm to −3.0 µm.

For TRPV2_RTx-ND_, 2254 movies were collected (30 frames/movie) with 60 s exposure and exposure rate of ~0.8 e^-^/pixel/s. The total exposure was of 42 e^-^/Å (Cao et al., 2013a) and a nominal defocus range from −1.25 µm to −3.0 µm.

### Reconstruction and refinement

*TRPV2_RTx-APOL_* MotionCor2 (Zheng et al., 2017) was used to perform motion correction and dose-weighting on 1293 movies. Unweighted summed images were used for CTF determination using GCTF (Zhang, 2016). Following motion correction and dose-weighting and CTF determination, micrographs which contained Figure of Merit (FoM) values of <0.12 and astigmatism values > 400 were removed, leaving 1207 micrographs for further analysis. An initial set of 1660 particles was picked manually and subjected to reference-free 2D classification (k = 12, T = 2) which was used as a template for automatic particle picking from the entire dataset (1207 micrographs). This yielded a stack of 580,746 particles that were binned 4 × 4 (4.64 Å/pixel, 64 pixel box size) and subjected to reference-free 2-D classification (k = 58, T = 2) in RELION 3.0 (Zivanov et al., 2018). Classes displaying the most well-defined secondary structure features were selected (470,760 particles) and an initial model was generated from the 2D particles using the Stochastic Gradient Descent (SGD) algorithm as implemented in RELION 3.0. 3D auto-refinement in RELION 3.0 was performed on the 470,760 particles with no symmetry imposed (C1), using the initial model, low-pass filtered to 30 Å, as a reference map. This resulted in an 8.9 Å 3D reconstruction, which was then used for re-extraction and re-centering of 2 × 2 binned particles (2.16 Å/pixel, 128 pixel box size). 3D classification (k = 4, T = 8) without imposed symmetry (C1) was performed on the extracted particles, using a soft mask calculated from the full molecule. Classes 2–4 (90,862, 109,623 and 101,570 particles, respectively) all possessed well-defined secondary structure, but visual inspection of the maps suggested that the classes represented distinct conformational states. Therefore, each class was processed separately. For each class, the particles were extracted and unbinned (1.08 Å/pixel, 256 pixel box size), and soft masks calculated. 3D auto-refinement of the individual classes without symmetry imposed (C1) yielded 4.7 Å (class 2), 3.6 Å (class 3) and 3.2 Å (class 4) 3D reconstructions. Inspection of these volumes revealed that classes 2 and 3 adopted two-fold (C2) symmetry, while class four was four-fold symmetric (C4). Particles from class 2 were subjected to particle movement and dose-weighting using the ‘particle polishing’ function as implemented in RELION 3.0. The shiny particles were input into 3D auto-refinement with a soft mask and C2 symmetry applied, resulting in a 4.19 Å reconstruction (TRPV2_RTx-APOL 3_). Similarly, particles from class 3 were subjected to polishing, and the following 3D auto-refinement with a soft mask and C2 symmetry applied resulted in a 3.3 Å final reconstruction (TRPV2_RTx-APOL2_). Particles from class 4 were first subjected to CTF refinement using the ‘CTF refine’ feature in RELION 3.0. Particle polishing was then performed, followed by 3D auto-refinement with a soft mask and C4 symmetry applied, yielding a 2.91 Å reconstruction (TRPV2_RTx-APOL 1_). All resolution estimates were based on the gold-standard FSC 0.143 criterion (Scheres and Chen, 2012; Chen et al., 2013).

*TRPV2_RTx-ND_* The 2254 collected movies were subjected to motion correction and dose-weighting (MotionCor2) and CTF estimation (GCTF) in RELION 3.0. Micrographs with FoM values < 0.13 and astigmatism values > 400 were removed, resulting in a selection of 1580 good micrographs. From these, 2015 particles were picked manually, extracted (without binning, 1.08 Å/pixel, 256 pixel box size) and subjected to reference-free 2D classification (k = 12, T = 2) that was used as a template for autopicking. This resulted in a 1,407,292 stack of particles that were binned 4 × 4 (4.32 Å/pixel, 64 pixel box size) and subjected to reference-free 2D classification (k = 100, T = 2). Classes exhibiting the most well-defined secondary structure features were selected, resulting in 482,602 particles. These were re-extracted (2 × 2 binned, 2.16 Å/pixel, 128 pixel box size) and put into 3D auto-refinement, using the previously obtained map of apo TRPV2 (EMD-6455) filtered to 30 Å as a reference with no symmetry applied (C1). The 3D auto-refinement yielded a 5.4 Å reconstruction. The particles were then subjected to 3D classification (k = 6, T = 8), with a soft mask and the 5.4 Å volume as a reference without imposed symmetry (C1). Only two of the six classes (classes 1 and 6) contained significant density in the TM domains. They were selected (112,622 particles), re-extracted, re-centered and unbinned (1.08 Å/pixel, 256 pixel box size) before being input into 3D auto-refinement without symmetry imposed (C1) and with a soft mask and the previous 5.4 Å reconstruction filtered to 30 Å as a reference. The 3D auto-refinement resulted in a 4.12 Å map, which was then subjected to Bayesian particle polishing. 3D auto-refinement was then performed on the resulting shiny particles with no symmetry applied (C1), resulting in a 4 Å reconstruction. The particles were then subjected to CTF refinement, yielding a 3D reconstruction resolved to 4 Å (C1). However, visual inspection of the map revealed a strong tendency towards two-fold symmetry. Therefore, 3D auto-refinement was repeated with C2 symmetry applied, resulting in a map resolved to 3.84 Å as estimated by gold-standard FSC 0.143 criterion.

### Model building

The TRPV2_RTx-APOL_ and TRPV2_RTx-ND_ models were built into the cryo-EM electron density map in Coot (Emsley and Cowtan, 2004), using the structures of TRPV2 (PDB 5AN8 and 6BWJ) as templates. The structures were real-space refined in Coot, and iteratively refined using the phenix.real_space_refine as implemented in the Phenix suite (Adams et al., 2010). Structures were refined using global minimization and rigid body, with high weight on ideal geometry and secondary structure restraints. The Molprobity server (Chen et al., 2010) (http://molprobity.biochem.duke.edu/) was used to identify problematic areas, which were subsequently manually rebuilt. The radius of the permeation pathways was calculated using HOLE (Smart et al., 1996). All analysis and structure illustrations were performed using Pymol (The PyMOL Molecular Graphics System, Version 2.0) and UCSF Chimera (Pettersen et al., 2004).

## Data Availability

The EM maps and atomic models have been deposited with the Electron Microscopy Data Bank (accession numbers EMD-20143, EMD-20145, EMD-20146, and EMD-20148) and the Protein Data Back (entry codes 6OO3, 6OO4, 6OO5, and 6OO7), respectively. The following datasets were generated: ZubcevicLHsuALBorgniaMJLeeS-Y2019Cryo-EM structure of the C4-symmetric TRPV2/RTx complex in amphipol resolved to 2.9 AElectron Microscopy Data BankEMD-20143 ZubcevicLHsuALBorgniaMJLeeS-Y2019Cryo-EM structure of the C2-symmetric TRPV2/RTx complex in amphipol resolved to 3.3 AElectron Microscopy Data BankEMD-20145 ZubcevicLHsuALBorgniaMJLeeS-Y2019Cryo-EM structure of the C2-symmetric TRPV2/RTx complex in amphipol resolved to 4.2 AElectron Microscopy Data BankEMD-20146 ZubcevicLHsuALBorgniaMJLeeS-Y2019Cryo-EM structure of the C2-symmetric TRPV2/RTx complex in nanodiscsElectron Microscopy Data BankEMD-20148 ZubcevicLHsuALBorgniaMJLeeS-Y2019Cryo-EM structure of the C4-symmetric TRPV2/RTx complex in amphipol resolved to 2.9 AProtein Data Bank6OO3 ZubcevicLHsuALBorgniaMJLeeS-Y2019Cryo-EM structure of the C2-symmetric TRPV2/RTx complex in amphipol resolved to 3.3 AProtein Data Bank6OO4 ZubcevicLHsuALBorgniaMJLeeS-Y2019Cryo-EM structure of the C2-symmetric TRPV2/RTx complex in amphipol resolved to 4.2 AProtein Data Bank6OO5 ZubcevicLHsuALBorgniaMJLeeS-Y2019Cryo-EM structure of the C2-symmetric TRPV2/RTx complex in nanodiscsProtein Data Bank6OO7

## Additional files

Transparent reporting form
